# Metabolic rewiring and autophagy inhibition correct lysosomal storage disease in mucopolysaccharidosis IIIB

**DOI:** 10.1016/j.isci.2024.108959

**Published:** 2024-01-29

**Authors:** Melania Scarcella, Gianluca Scerra, Mariangela Ciampa, Marianna Caterino, Michele Costanzo, Laura Rinaldi, Antonio Feliciello, Serenella Anzilotti, Chiara Fiorentino, Maurizio Renna, Margherita Ruoppolo, Luigi Michele Pavone, Massimo D’Agostino, Valeria De Pasquale

**Affiliations:** 1 Department of Molecular Medicine and Medical Biotechnology, University of Naples Federico II, Via S. Pansini 5, 80131 Naples, Italy; 2 CEINGE Biotecnologie Avanzate Franco Salvatore, Via G. Salvatore 486, 80131 Naples, Italy; 3 Department of Science and Technology, University of Sannio, Via F. de Sanctis, 82100 Benevento, Italy; 4 Department of Veterinary Medicine and Animal Productions, University of Naples Federico II, Via F. Delpino 1, 80137 Naples, Italy

**Keywords:** Human metabolism, Cell biology

## Abstract

Mucopolysaccharidoses (MPSs) are lysosomal disorders with neurological involvement for which no cure exists. Here, we show that recombinant NK1 fragment of hepatocyte growth factor rescues substrate accumulation and lysosomal defects in MPS I, IIIA and IIIB patient fibroblasts. We investigated PI3K/Akt pathway, which is of crucial importance for neuronal function and survival, and demonstrate that PI3K inhibition abolishes NK1 therapeutic effects. We identified that autophagy inhibition, by Beclin1 silencing, reduces MPS IIIB phenotype and that NK1 downregulates autophagic-lysosome (ALP) gene expression, suggesting a possible contribution of autophagosome biogenesis in MPS. Indeed, metabolomic analyses revealed defects of mitochondrial activity accompanied by anaerobic metabolism and inhibition of AMP-activated protein kinase (AMPK), which acts on metabolism and autophagy, rescues lysosomal defects. These results provide insights into the molecular mechanisms of MPS IIIB physiopathology, supporting the development of new promising approaches based on autophagy inhibition and metabolic rewiring to correct lysosomal pathology in MPSs.

## Highlights

• Recombinant NK1 rescues lysosomal pathology in MPS I, IIIA, and IIIB fibroblasts
• PI3K/Akt activation pathway is fundamental for NK1 therapeutic effects
• Metabolic rewiring through AMPK inhibition rescues lysosomal pathology in MPS IIIB
• Autophagy inhibition by Beclin1 silencing rescues lysosomal pathology in MPS IIIB

## Graphical abstract

**Figure:**
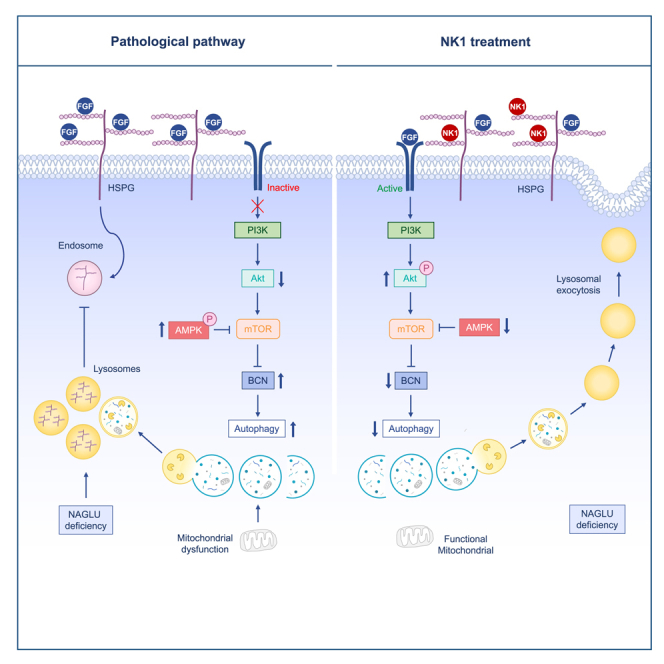

## Introduction

Mucopolysaccharidoses (MPSs) are a family of inherited lysosomal storage disorders caused by mutations of genes coding for lysosomal enzymes essential in the metabolic degradation of glycosaminoglycans (GAGs).^1^ Depending on the type of undegraded or partially degraded GAG which accumulates, MPSs are classified in seven types: MPS I (Hurler, Scheie, and Hurler-Scheie syndromes) and MPS II (Hunter syndrome) with heparan sulfate (HS) and dermatan sulfate (DS) being the accumulated substrates, MPS III (Sanfilippo syndrome) where HS is the only stored GAG, MPS IV (Morquio syndrome) with keratan sulfate (KS) and chondroitin sulfate (CS) accumulation, MPS VI (Maroteaux-Lamy syndrome) with DS buildup, MPS VII (Sly syndrome) where HS, DS, and CS gather, and MPS IX where hyaluronan (HA) accumulates.^1^ Lysosomal accumulation of GAGs results in cell, tissue, and organ dysfunctions leading to chronic and progressive disorders with a wide range of clinical symptoms, although in variable degree.^2^^,^^3^

Among the distinct types of MPSs, we focused our research efforts toward a better understanding of the physiopathology of MPS IIIB subtype (OMIM # 252920), and the development of a new therapeutic approach for this disease that is still without any available disease modifying treatments.^2^^,^^4^^,^^5^^,^^6^^,^^7^^,^^8^^,^^9^^,^^10^^,^^11^^,^^12^ Patients affected by MPS IIIB develop progressive and severe neurological disorders and other somatic manifestations, and usually die during the second or third decade of life.^13^^,^^14^ The disease is caused by autosomal recessive defects of the α-N-acetylglucosaminidase (NAGLU, EC:3.2.1.50) enzyme required for HS degradation, thus leading to the accumulation of HS, and HS-derived oligosaccharides, in tissue and organs including the central nervous system (CNS) which is particularly sensitive to this metabolic derangement.^1^ Interestingly, our investigations on MPS IIIB pathogenic mechanisms highlighted that, in cells of affected patients, HS storage is not restricted to the lysosomal compartment, but it is also redistributed to different cellular and extracellular localizations, as also demonstrated for other MPSs.^15^^,^^16^^,^^17^^,^^18^ On the cell surface and within the extracellular matrix of cells, HS chains are covalently bound to a protein core, forming the so-called HS proteoglycans (HSPGs) which play a fundamental role in growth factor receptor activation and signaling, among many other regulatory activities.^19^^,^^20^^,^^21^^,^^22^ Therefore, a perturbation of HSPG-dependent signaling and related cellular processes has been associated with several human diseases, including MPSs.^2^^,^^17^^,^^23^^,^^24^^,^^25^ Cell signaling through fibroblast growth factor-2 (FGF2) is one of these fundamental processes to achieve typical neurodevelopment. FGF2 is a key neurotrophic factor that plays a role in early neural induction, CNS patterning (e.g., neural plate formation) and the development of functional neural circuits, such as the spinal cord and neocortex.^26^ As a result, FGF2 coordinates hippocampal neurogenesis, synaptic growth and formation, all of which regulate learning, memory, and injury response.^26^ Of note, there is a significant impairment in FGF2 signaling in MPS IIIB due to excess extracellular HSPG accumulation; likely contributing to the early neurobehavioral phenotype seen in affected individuals.^2^^,^^7^ On these bases, we have developed an innovative approach for the treatment of the cellular signaling and metabolic defects of individuals with MPS IIIB that uses a recombinant protein targeting HSPGs on the cell surface and in the extracellular matrix.^4^^,^^7^

Indeed, we explored the potential therapeutic efficacy of recombinant hepatocyte growth factor/scatter factor (HGF/SF) natural spliced variant NK1, which binds HS with high affinity.^27^^,^^28^ We demonstrated that NK1 is capable of reducing HS accumulation and lysosomal pathology in MPS IIIB patient-derived fibroblasts by restoring FGF2 signaling. We also demonstrated that NK1 is effective in rescuing the morphological and functional dysfunctions of lysosomes in a neuronal cellular model of the MPS IIIB generated by our team.^4^ These encouraging results prompted us to investigate the molecular mechanism of action of NK1 at the cellular and molecular level, using both cellular tools and brain tissues from the mouse model of the MPS IIIB disease.^29^

Emerging evidence suggests that multiple factors may contribute to the progression of lysosomal storage diseases.^12^^,^^30^^,^^31^^,^^32^^,^^33^^,^^34^ In this study, we investigated the activity of NK1 on the main molecular pathways involved in cell signaling, metabolism, and autophagy whose dysregulation is known to contribute to the pathogenesis of MPS IIIB.^6^^,^^7^^,^^9^^,^^10^^,^^30^^,^^35^^,^^36^ Most growth factors act through tyrosine kinase receptors and subsequently activate two main pathways, namely MEK/ERK and PI3K/Akt. Therefore, we performed additional testing to determine to which of these critical pathways is involved in the beneficial effect of NK1 in MPS IIIB patient-derived fibroblasts. Our results demonstrate that NK1 is able to reduce lysosomal pathology in our disease specific fibroblast cell model via activation of the PI3K/Akt signaling pathway. As this pathway is known to modulate/repress autophagy,^37^^,^^38^ we investigated whether the mechanism of action of NK1 in rescuing MPS related lysosomal pathology might involve autophagy regulation. Interestingly, while it has been well established that autophagy processes can be impaired in MPSs, contradictory results on autophagy mechanisms and therapeutic potential of autophagy activators/inhibitors have been reported in the literature.^39^ Indeed, while Vitry et al.^40^ observed normal function of the autophagy pathway in neurons of MPS IIIB mouse model, we found impaired lysosomal autophagy mechanisms in liver and heart of NAGLU^−/−^ mice.^5^^,^^9^ Here, we demonstrate that NK1 affects autophagy mechanisms by downregulating the expression of autophagic-lysosome (ALP) genes allowing an improvement of the disease phenotype. Finally, a targeted metabolomic approach coupled with a Seahorse analysis of the oxygen consumption rate (OCR) profile of a NAGLU silenced neuroblastoma cell line, allowed us to detect an abnormal mitochondrial function associated with anaerobic glycolytic metabolism in our MPS IIIB model system.

## Results and discussion

### NK1 rescues HS accumulation and lysosomal defects in MPS I, IIIA, and IIIB patient-derived fibroblasts

**Figure 1:**
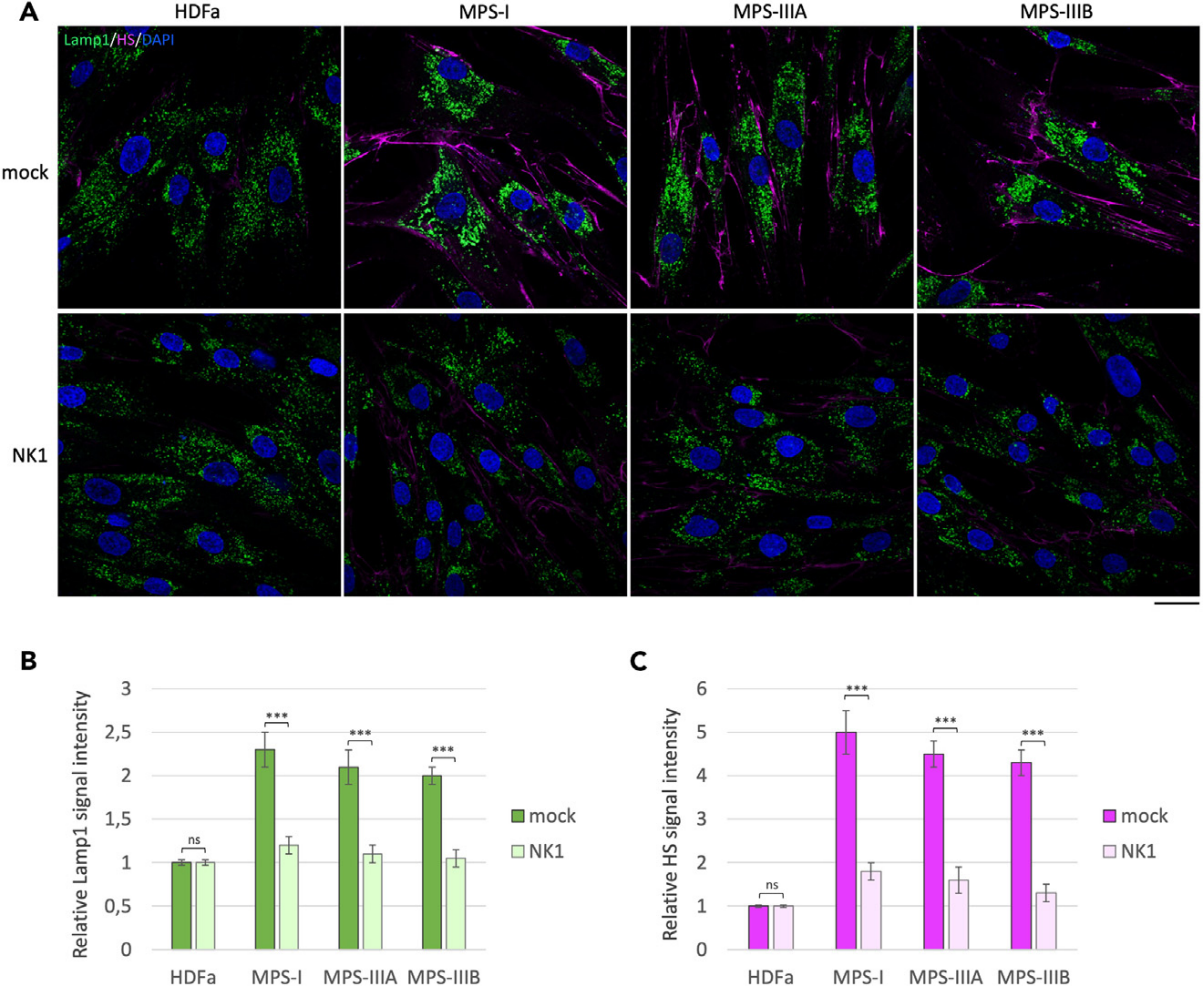
NK1 rescues lysosomal defects and HS accumulation in MPS I, IIIA, and IIIB patient fibroblasts (A) Human dermal fibroblasts (HDFa) from a healthy control or MPS I, MPS IIIA, and MPS IIIB patient fibroblasts were grown on coverslips and treated or not (mock) with NK1 10^−6^ M for 48 h before being processed for indirect immunofluorescence. The lysosomal marker LAMP1 (green) and heparan sulfate (pink) were revealed by using specific antibodies. Nuclei (blue) were decorated by DAPI staining. Single focal sections are shown. Images are representative of three independent experiments made in triplicates. Scale bar: 50 μm. (B and C) Histograms show the quantification relative to the percentage of cells with pathological enlarged lysosomes (green bars) and HS mean value of fluorescence intensity (pink bars). Means ± SEM were obtained from three independent experiments. ∗∗∗ p value <0.001. ns = not significant.

The lack of lysosomal HS digestion in most MPSs causes the accumulation of enlarged lysosomal vacuoles within the cytoplasm of the cells and an abnormal buildup of HSPGs on the cell membrane.^2^^,^^4^^,^^7^^,^^41^ In order to evaluate whether NK1 treatment would be able to reduce HS accumulation and lysosomal phenotype in fibroblasts derived from MPS patients with genetic defects of enzymes involved in HS degradation, we performed co-immunofluorescence experiments by staining MPS I, IIIA, and IIIB patient-derived fibroblasts and human dermal adult fibroblasts (HDFa) as control with specific antibodies against LAMP1, for lysosomal staining, and HS (10E4 clone). The 10E4 anti-HS antibody is specific for the recognition of HS accumulated on cell membranes, as the epitope of the antibody is localized in the N-sulfated regions of HS chains.^42^ A strong accumulation of HS (pink color) on the cell membrane and a pathological centromeric localization of enlarged lysosomes (green color) were observed in MPS patient-derived fibroblasts as compared to HDFa control fibroblasts (Figure 1).

Upon treatment with the recombinant NK1 protein, at the concentration of 10^−6^ M for 48 h, cells displayed a reduced accumulation of HS on the cell membrane. Moreover, NK1 treatment also rescued the morphological lysosomal defects featuring the pathological lysosomes of MPS I, IIIA, and IIIB fibroblasts, such as the enlarged sized and the centromeric perinuclear distribution. No effects were detected after treatment of control HDFa with NK1. These findings confirm our previous results obtained from NK1-treated fibroblasts of MPS I and MPS IIIB affected patients,^7^ and demonstrate for the first time a similar beneficial effect of NK1 either on the pathological accumulation of HS on the cell membrane or the morphological lysosomal defects in fibroblasts derived from MPS IIIA affected patients. Furthermore, the results shown in Figure 1 indicate that NK1 treatment also has an important effect on the regulation of lysosome trafficking, because the cellular distribution of these organelles, after cell exposure to NK1, is no longer centromeric, but it appears redistributed throughout the cytoplasm of the cells. This result is consistent with our previous findings showing that NK1 is able to reactivate the physiological lysosomal exocytosis and secretion in a cellular model of MPS IIIB.^4^

### Inhibition of PI3K pathway abrogates NK1 effects on lysosomal pathology in MPS I, IIIA, and IIIB patient-derived fibroblasts

Our previous studies suggested that the therapeutic efficacy of NK1 consists in masking the excess of HS accumulated on cell membrane with the consequent restoration of the physiological balance between HSPGs, morphogens or growth factors and their cognate receptors.^7^ Most of the growth factors act through tyrosine kinase receptors which in turn activate two main pathways: MEK/ERK and PI3K/Akt.^43^ Therefore, using two specific inhibitors, we tested whether and how the response of patient-derived fibroblasts to NK1 treatment might involve these signaling pathways.

**Figure 2:**
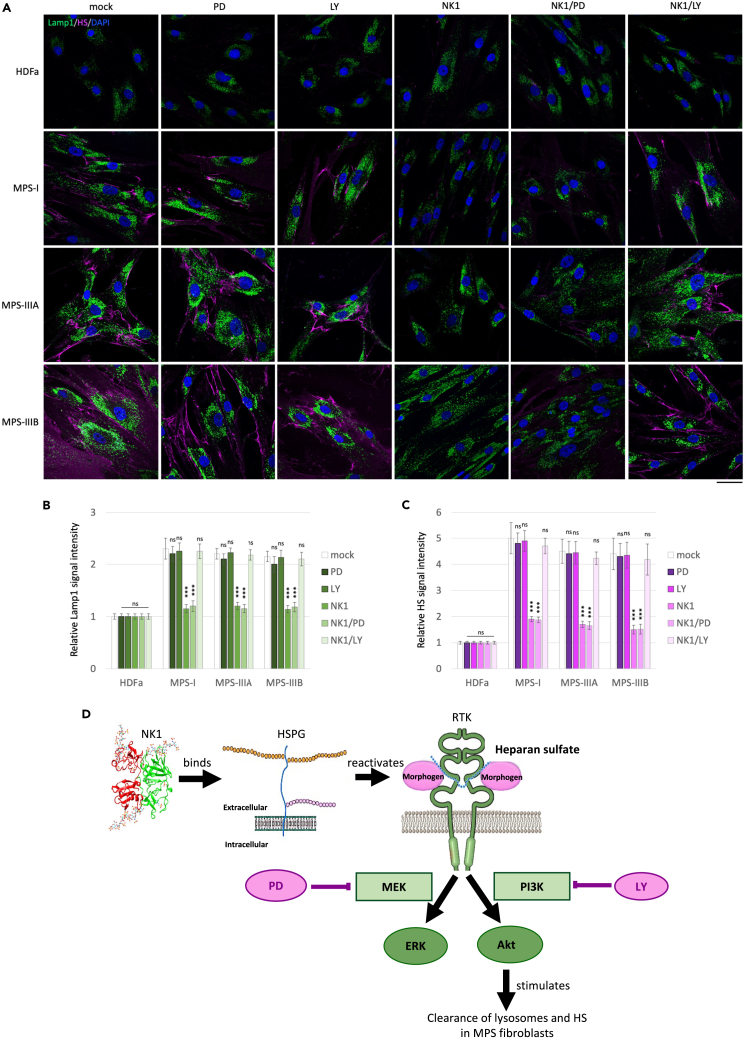
NK1 therapeutic efficacy is abrogated by PI3K inhibition (A) Human dermal adult fibroblasts (HDFa) or MPS I, MPS IIIA, and MPS IIIB patient fibroblasts were grown on coverslips and treated or not with MEK/ERK pathway inhibitor (PD98059) for 90′ at the concentration of 5x10^−5^ M, PI3K/Akt pathway inhibitor (LY294002) for 60′ at the concentration of 5x10^−5^ M, NK1 recombinant protein for 48 h at the concentration of 10^−6^ M, and the combination NK1/PD98059 or NK1/LY294002 before being processed for indirect immunofluorescence. The lysosomal marker LAMP1 (green) and HS (pink) were revealed by using specific antibodies. Nuclei (blue) were decorated by DAPI staining. Single focal sections are shown. Images are representative of three independent experiments made in triplicates. Scale bar: 50 μm. (B and C) Histograms show the quantification relative to the percentage of cells with pathological enlarged lysosomes (green bars) and HS mean value of fluorescence intensity (pink bars). Means ± SEM were obtained from three independent experiments. ∗∗∗ p value <0.001. ns = not significant. (D) Scheme of the molecular mechanism of action of NK1 which goes through HS binding, restoration of endogenous growth factor signaling, and PI3K/Akt activation rather than MEK/ERK pathway.

HDFa control and MPS patient-derived fibroblasts were independently treated with the MEK/ERK inhibitor PD98059^6^^,^^43^ or the PI3K/Akt inhibitor LY294002.^43^ Treated fibroblasts were subjected to immunofluorescence analysis, monitoring both LAMP1 and HS signal intensity. Treatment with both inhibitors had no effect on HS accumulation and lysosomal defects in control and patient-derived fibroblasts as compared to untreated fibroblasts (Figure 2A). Whereas, after treatment with NK1, at a concentration of 10^−6^M for 48 h, cells displayed a reduced the HS accumulation and the recovery of normal lysosomal morphology and distribution. The combined pretreatment with PD98059 and treatment with NK1 did not affect the beneficial effects of NK1 in MPS-affected fibroblasts, thus demonstrating that NK1 functional activity is not mediated by the MEK/ERK pathway activation. By contrast, the combined treatment with LY294002 and NK1 abolished the beneficial action of NK1 on the MPS I, IIIA, and IIIB patient-derived fibroblasts. These results suggest that NK1 treatment could exert its therapeutic action in reducing HS accumulation and lysosomal defects in MPS fibroblasts through the activation of the PI3K/Akt pathway rather than MEK/ERK pathway (Figure 2B).

Western blotting analysis (Figure S1) confirmed the reduced ERK phosphorylation in MPS I, IIIA, and IIIB fibroblasts upon treatment with the MEK/ERK pathway inhibitor PD98059 in combination with NK1 as compared to the NK1 treated alone. On contrary, upon LY294002 treatment in combination with NK1, *p*-ERK levels remained unchanged as compared to the NK1 treated alone.

### Autophagy inhibition through Beclin1 (*BCN1*) silencing rescues lysosomal defects in MPS IIIB model systems

**Figure 3:**
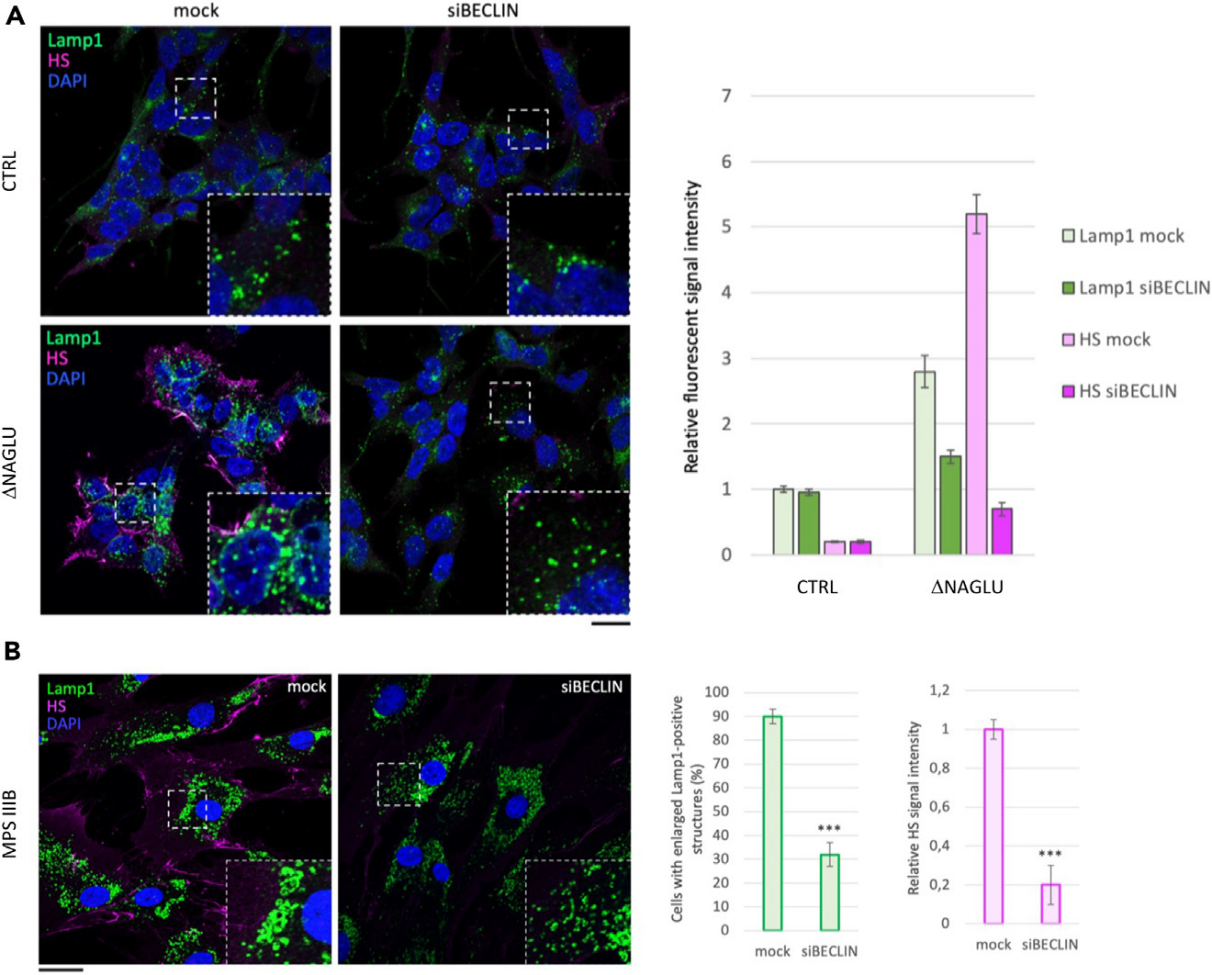
Autophagy inhibition through Beclin1 (*BCN1*) silencing rescues lysosomal defects and HS accumulation in MPS IIIB model systems (A) SK-NBE (CTRL) and ΔNAGLU clones were grown on coverslips and silenced for Beclin-1 by siRNA for 24 h before being processed for indirect immunofluorescence. The lysosomal marker LAMP1 (green) and heparan sulfate (pink) proteins were revealed by using specific antibodies. Nuclei (blue) were decorated by DAPI staining. Single focal sections are shown. Images are representative of three independent experiments made in triplicates. Scale bar: 50 μm. (B) MPS IIIB fibroblasts were treated as in A. Single focal sections are shown. Images are representative of three independent experiments made in triplicates. Scale bar: 50 μm. The histograms on the right for (A) and (B) show the quantification relative to the percentage of cells with pathological enlarged lysosomes (green bars) and HS mean value of fluorescence intensity (pink bars). Means ± SEM were obtained from three independent experiments. ∗∗∗ p value <0.001.

On the basis of the previously described results, indicating that NK1 is able to reduce lysosomal pathology in MPS patient-derived fibroblasts via activation of PI3K/Akt signaling pathway, which is known to modulate/repress autophagy,^37^ we tested whether interfering with autophagosome formation could mimic the effects of NK1 treatment. To this purpose, Beclin1 (*BCN1*) gene expression was knocked down in the NAGLU-silenced human neuroblastoma SK-NBE (ΔNAGLU clones), a neuronal cellular model of MPS IIIB generated in our laboratory,^4^ ΔNAGLU clones were treated for 24 h with a specific siRNA against *BCN1*, a key effector of early stage autophagosome formation process,^44^ and processed for LAMP1 and HS immunofluorescence. In ΔNAGLU clones, a strong accumulation of the HS (pink color) on cell membrane and abnormal centromeric localization of enlarged lysosomes (green color) was observed, compared to control SK-NBE transfected with a scrambled shRNA (CTRL). *BCN1* silencing in ΔNAGLU clones triggered a strong reduction of HS accumulation and lysosomal vacuolization indicating that the inhibition of autophagy would exert a favorable action, since less cargo would be carried to the already unfunctional lysosomes (Figure 3A). The efficacy of autophagy inhibition on lysosomal properties was also confirmed by confocal microscopy analysis of MPS IIIB patient-derived fibroblasts either untreated or treated with *BCN1* siRNA. The results obtained showed a significant reduction of both HS accumulation and lysosomal pathology in the MPS IIIB patient-derived fibroblasts silenced for *BCN1* as compared to parental cells (Figure 3B).

### The mechanism of action of NK1 in rescuing lysosomal pathology involves autophagy inhibition

**Figure 4:**
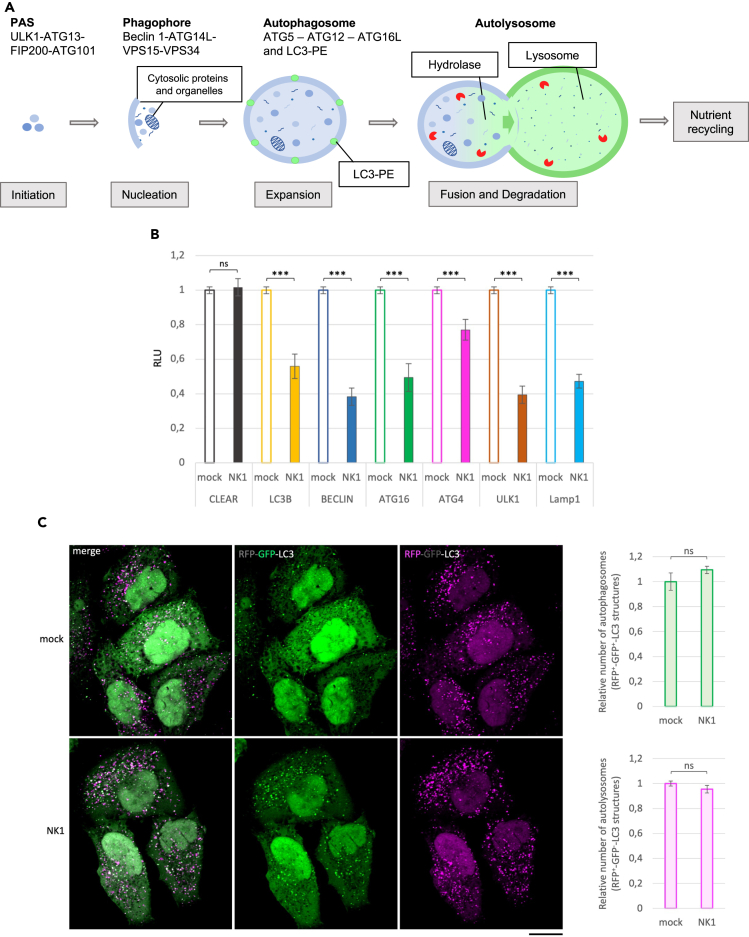
Inhibitory effects of NK1 on the autophagic mechanisms (A) Schematic representation of the key players in the autophagic processes. (B) Luciferase assay on HeLa cells treated with NK1. The activation of several autophagic genes before and after NK1 treatment was measured by a luciferase assay. Notably, each vector codifies for the Firefly luciferase whose expression is controlled by the different autophagic gene promoters. The Relative LUciferase intensity (RLU) between mock and NK1 treated HeLa cells is represented by histograms. Means ± SEM were obtained from three independent experiments. ns = not significant. ∗∗∗ p value <0.001. (C) Traffick-light HeLa cells stably expressing RFP-GFP-LC3 were grown on coverslip for 24 h before being treated or not with NK1 for 24 h at the concentration of 10^−6^ M. After treatment, cells were employed for immunofluorescence and confocal microscopy analysis. Single focal sections are shown. Images are representative of three independent experiments made in triplicates. Scale bar: 50 μm. The histograms on the right report the relative number of autophagosomes (RFP^+^-GFP^+^-LC3 structures, green bars) and of autolysosomes (RFP^+^-GFP^-^-LC3 structures, pink bars). Means ± SEM were obtained from three independent experiments. ns = not significant.

The observed PI3K/Akt pathway-dependence of NK1 action and the known link between this signaling pathway and autophagosome formation inhibition^45^ prompted us to investigate whether NK1 could exert its corrective effect through autophagy regulation. To this aim, we tested first whether NK1 treatment could influence autophagic gene expression by transiently over-expressing in HeLa cells different vectors bearing luciferase gene downstream of the promoter regions of the ALP genes *LC3*, *BCN1*, *ATG16*, *ATG4*, *ULK1*, *LAMP1*, and downstream a DNA response element motif known as the coordinated lysosomal expression and regulation (CLEAR) element to which the microphthalmia-transcription factor E family (MiT), including transcription factor EB (TFEB) and TFE3 bind.^46^^,^^47^^,^^48^^,^^49^ The involvement of the encoded proteins in the autophagy is summarized in Figure 4A. Compared to untreated HeLa cells, NK1 treatment caused a significant reduction of luciferase activity, suggesting a reduced expression of all tested ALP genes, besides the *CLEAR* element (Figure 4B). These results indicate a transcriptional control of the main ALP genes by NK1, thereby indicating an upstream control of the ALP possibly independent from TFEB and TFE3 action.

Moreover, we tested whether NK1 could also exert a downstream influence on the autophagic pathway. To this aim, we used the traffic-light system^50^ that exploits the high sensitivity at low pH of a green fluorescent protein (GFP) fused in tandem with a pH-insensitive red fluorescent protein (RFP) at the N-terminus of the autophagic marker LC3. Notably, during autophagosome formation, the neutral pH of the cytoplasm renders both GFP and RFP fluorescent. Subsequently, upon fusion with lysosomes, the acidity of the autolysosome lumen keeps RFP fluorescent while switching off the GFP signal. As shown in Figure 4C, NK1 treatment did not affect either the number of RFP^+^-GFP^+^-LC3 positive autophagosomes (upper histogram in green) or the number of RFP^+^-GFP^-^-LC3 positive autolysosomes (lower histogram in magenta).

Overall, these findings suggest that NK1 could act as an upstream down-modulator of the autophagic pathway by reducing the gene expression of several critical components of autophagosome biogenesis.

### Metabolomic analyses of MPS IIIB cell and mouse model systems show accumulation of metabolites related to an impairment of mitochondrial function

To further investigate the molecular mechanisms of the MPS IIIB physiopathology, a metabolome analysis of NAGLU-silenced human SK-NBE clone (ΔNAGLU) was performed in comparison to a control clone (CTRL) using a targeted metabolomic approach. A comprehensive list of the measured metabolites, including metabolite names and their raw concentrations in each biological replicate, is shown in Table S1.

**Figure 5:**
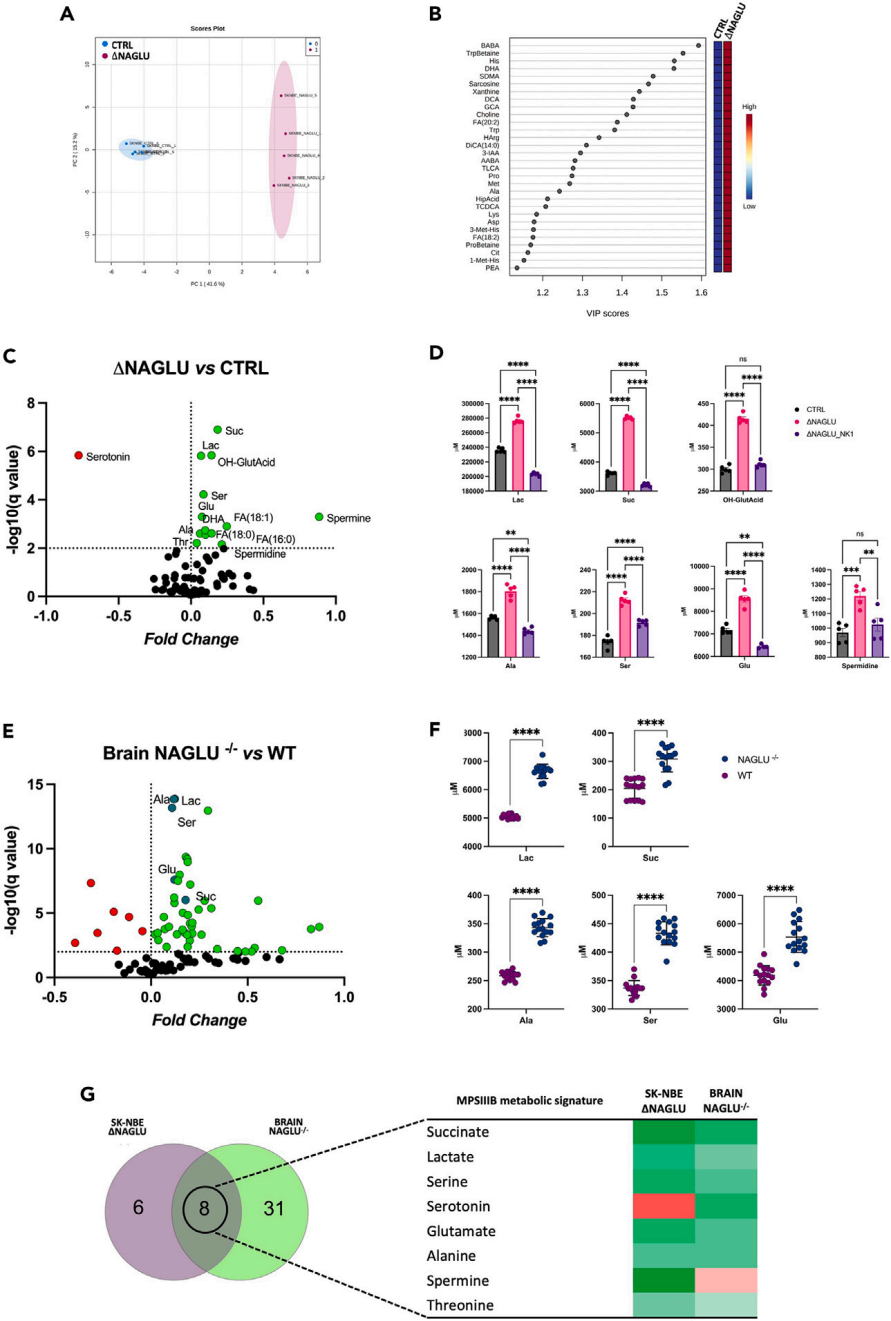
Descriptive comparative analysis of the metabolome of MPS IIIB system models (A) PCA was performed using cellular metabolites levels from ΔNAGLU in respect to CTRL according to PC1 41.6% and PC2 15.2%. (B) The 30 discriminant features identified with values of VIP (Variable Importance in Projection) scores >1.0 are reported. (C and E) Volcano plot analysis of significantly different metabolites in ΔNAGLU vs. CTRL clones, and NAGLU^−/−^ vs. WT mouse brains. The green and red dots represent the increased and decreased metabolites, respectively. The dark green dots represent the common metabolites, found to be increased in both MPS IIIB system models. Black dots refer to all the metabolites identified in the dataset whose relative abundances are not significantly different between groups. (D) The abundances of succinate, lactate, 3-hydroxyglutarate, alanine, serine, glutamate and spermidine in ΔNAGLU clone were compared to CTRL clone and ΔNAGLU clone treated with NK1 (ΔNAGLU_NK1). Plots represent the analytes concentrations (means ± SEM). The significant differences between groups were evaluated performing ordinary one-way ANOVA test and Hold-Sidak’s multiple comparison test in normally distributed datasets or Kruskal-Wallis test and Dunn’s multiple comparison test in non-normally distributed datasets. The normal distribution was verified according to D'Agostino and Pearson tests. (∗p < 0.05, ∗∗p < 0.01, ∗∗∗p < 0.001 ∗∗∗∗p < 0.0001, ns = not significant). (F) The abundances of succinate, lactate, alanine, serine, and glutamate in brain tissue from NAGLU^−/−^ mice were evaluated in respect to WT. Plots represent the analytes concentrations (means ± SEM). The significant differences between groups were evaluated performing parametric t test with Welch correction in normally distributed datasets or Mann-Whitney test in non-normally distributed datasets (∗p < 0.05, ∗∗p < 0.01, ∗∗∗p < 0.001 ∗∗∗∗p < 0.0001, ns = not significant). (G) Eulero-Venn analysis of the differentially abundant metabolites in cellular (ΔNAGLU) and animal (NAGLU^−/−^ mice) models of MPS IIIB, focusing on common molecules.

Univariate and multivariate statistical analyses were employed for selecting the most significant metabolic alterations in ΔNAGLU with respect to CTRL clones. Specifically, the different rate and variances occurring between the groups were evaluated according to a principal component analysis (PCA), revealing a clear separation as reported by a PC1 variance of 41.6% and PC2 variance of 15.2% (Figure 5A). The Variable Importance in Projection (VIP) measure was used to identify the most discriminant hits of ΔNAGLU (Figure 5B). In particular, the levels of metabolites such as β-aminobutyric acid (BABA), TrpBetaine, His, and Docosahexaenoic acid (DHA) are useful to strongly discriminate (VIP>1.5), highlighting metabolic abnormalities likely associated with the MPS IIIB phenotype.

Interestingly, the hierarchical clustering of identified and quantified metabolites shown in the heatmap (Figure S2) exhibits a clear distinct pattern in the metabolite abundance between ΔNAGLU and CTRL. Univariate statistical analysis highlighted significant quantitative alterations in NAGLU-silenced clone, as shown in the volcano plot (Figure 5C). The metabolites showing significant differences between the two groups are listed in Table 1. In addition, we analyzed the degree of normalization upon NK1 treatment of these metabolite derangements that were identified in our MPS IIIB cell model system. Surprisingly, the cellular abundance of most of the metabolites accumulated in the MPS IIIB cell system, such as succinate, lactate, 3-hydroxyglutarate, alanine, serine, glutamate, and spermidine were normalized after NK1 treatment (Figure 5D). The differentially abundant metabolites in ΔNAGLU that did not revert, their levels are reported in Figure S3.

**Table 1:** Differentially abundant metabolites (μ M) in ΔNAGLU compared to CTRL

*Metabolite*	p *value*	*q value*	*Mean* ΔN*AGLU*^*−/−*^	*Mean CTRL*	*Difference*	*Fold Change*
Suc	<0.000001	<0.000001	5513.0	3614.0	1899.0	0.18
OH-GlutAcid	<0.000001	0.0000	415.2	300.0	115.2	0.14
Lac	<0.000001	0.0000	276651.0	235999.0	40652.0	0.07
Ser	0.0000	0.0000	212.3	174.0	38.3	0.09
Serotonin	0.0000	0.0003	29.7	176.8	−147.1	−0.77
DHA	0.0001	0.0010	93.2	74.5	18.7	0.10
Glu	0.0001	0.0010	8549.0	7175.0	1374.0	0.08
FA(18:1)	0.0001	0.0010	6243.0	3541.0	2701.0	0.25
Spermidine	0.0004	0.0031	1220.0	968.8	251.1	0.10
Ala	0.0006	0.0036	1804.0	1562.0	242.2	0.06
FA(16:0)	0.0006	0.0036	31029.0	19138.0	11891.0	0.21
Spermine	0.0007	0.0042	470.4	60.0	410.4	0.89
Thr	0.0008	0.0042	295.5	269.8	25.7	0.04
FA(18:0)	0.0011	0.0055	31820.0	22876.0	8945.0	0.14

See also Figure S3.

To better characterize the revealed signature, the metabolome of brains from 8 months old MPS IIIB mice was also investigated. Significant differentially abundant metabolites in NAGLU^−/−^ mice are shown in Figure 5E and listed in Table 2. In detail, the increased abundance of lactate, succinate, alanine, serine, and glutamate was validated in the brain of NAGLU^−/−^ mice as compared to age-matched wild-type mice (WT) (Figure 5F). The other significant metabolites of the MPS IIIB brain are reported in Figure S4.

**Table 2:** Differentially abundant metabolites (μ M) in Brain_NAGLU^−/−^ compared to WT

*Metabolite*	p *value*	*q value*	*Mean of NAGLU*^−/−^	*Mean of WT*	*Difference*	*Fold change*
Lac	<0.000001	<0.000001	6646	5058	1588	0.12
Ala	<0.000001	<0.000001	343.3	258.5	84.8	0.12
Ser	<0.000001	<0.000001	433.2	336.8	96.33	0.11
Serotonin	<0.000001	<0.000001	2.752	1.389	1.363	0.30
Cit	<0.000001	<0.000001	8.766	5.77	2.996	0.18
Pro	<0.000001	<0.000001	58.24	37.44	20.8	0.19
Lys	<0.000001	<0.000001	97.74	63.06	34.68	0.19
Ile	<0.000001	<0.000001	21.55	15.63	5.918	0.14
Glu	<0.000001	<0.000001	5535	4185	1350	0.12
3-Met-His	<0.000001	<0.000001	0.9482	0.6723	0.2759	0.15
GABA	<0.000001	<0.000001	0.2238	0.4582	−0.2344	−0.31
Suc	<0.000001	<0.000001	308	204.8	103.3	0.18
Orn	<0.000001	<0.000001	4.208	2.61	1.599	0.21
Val	0.000001	0.000005	50.42	38.03	12.38	0.12
HipAcid	0.000001	0.000005	0.1746	0.2685	−0.0939	−0.19
Spermine	0.000007	0.00002	5.956	7.71	−1.754	−0.11
H1	0.000007	0.00002	225.1	168.9	56.17	0.12
Creatinine	0.00003	0.000084	98.01	83.85	14.15	0.07
Gln	0.000034	0.000087	7761	3572	4189	0.34
beta-Ala	0.000088	0.000203	0.947	0.6352	0.3118	0.17
SDMA	0.000115	0.000255	0.0447	0.0288	0.0159	0.19
Choline	0.000128	0.000263	106.2	117.4	−11.18	−0.04
PAG	0.000149	0.000296	0.0214	0.05213	−0.03073	−0.39
Gly	0.000164	0.000314	810.3	682.6	127.7	0.07
Trp	0.000177	0.000328	9.572	6.49	3.082	0.17
Ind-SO4	0.000195	0.000349	7.424	5.941	1.483	0.10
Phe	0.000215	0.000374	40.63	23.8	16.84	0.23
Betaine	0.000238	0.000401	11.53	10.64	0.8827	0.03
Arg	0.000325	0.000531	75.59	72.01	3.577	0.02
CDCA	0.000356	0.000565	0.564	0.3304	0.2336	0.23
Tyr	0.000833	0.00125	38.65	19.58	19.07	0.30
Trigonelline	0.001036	0.001514	0.9762	0.6248	0.3515	0.19
Thr	0.001216	0.001732	133	121.5	11.44	0.04
TMAO	0.001742	0.002419	0.9074	0.553	0.3544	0.22
Sarcosine	0.002007	0.002697	1.446	0.9229	0.5235	0.20
BABA	0.00275	0.003472	0.5718	0.9282	−0.3565	−0.21
AABA	0.003299	0.004072	1.419	1.163	0.2564	0.09
Met-SO	0.007729	0.009334	0.3489	0.1544	0.1945	0.35
Leu	0.008154	0.009638	28.88	21.29	7.599	0.13

See also Figure S4.

The differential metabolomes of ΔNAGLU cells and NAGLU^−/−^ brains were analyzed to retrieve the relevant metabolic networks and pathways enriched in each condition (Figures S5 and S6).

The metabolic signature shared between both MPS IIIB model systems globally includes a group of eight metabolites (Figure 5G). The analysis showed 8 metabolites as being in common for both conditions. Among them 6 metabolites showed the same trend of abundance throughout the replicates such as succinate, lactate, serine, glutamate, alanine, and threonine that were upregulated in both cell and murine MPS IIIB models as compared to their controls. On the other hand, serotonin and spermine had a different trend among the MPS IIIB models.

### Metabolic analyses unravel an anaerobic glycolytic metabolism associated with an impairment of mitochondrial activity in MPS IIIB cellular model system

**Figure 6:**
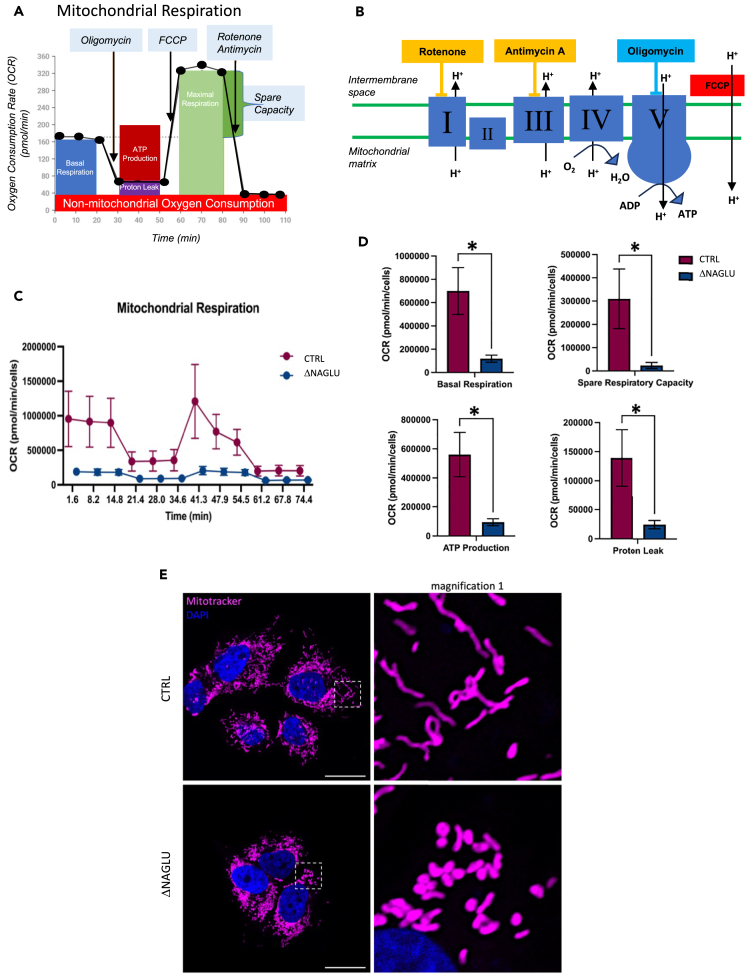
Metabolic profile and mitochondrial activity/morphology in MPS IIIB cell model system (A) OCR profile demonstrating measurement of associated parameters of mitochondrial respiration (modified from Agilent Seahorse XF Cell Mito Stress Test Kit brochure). (B) Scheme of the modulators of mitochondrial electron transport chain used to determine the bioenergetic parameters (modified from Agilent Seahorse XF Cell Mito Stress Test Kit brochure). (C) Real-time oxygen consumption rate (OCR) of NAGLU silenced SK-NBE (ΔNAGLU) compared to control clone (CTRL) was measured at 37°C using a Seahorse XF Analyzer (Seahorse Bioscience, North Billerica, MA, USA). Clones were plated into specific cell culture microplates (Agilent, USA) at the concentration of 3x10^4^ cells/well, and cultured for 12 h in DMEM, 10% FBS. OCR was measured in XF media (non-buffered DMEM medium, containing 10 mM glucose, 2 mM L-glutamine, and 1 mM sodium pyruvate) under basal condition and after sequential addition of 1.5 μM oligomycin, 2 μM FCCP, and rotenone + antimycin (0.5 μM all) (all from Agilent). Reported data are the means ± SEM of three measurements. (D) Indices of mitochondrial respiratory function were calculated from OCR profile: basal OCR (before addition of oligomycin), maximal respiration (calculated as the difference between FCCP rate and antimycin + rotenone rate), spare respiratory capacity (calculated as the difference of FCCP-induced OCR and basal OCR), ATP production (calculated as difference between basal OCR and oligomycin-induced OCR) and proton leak (calculated as the difference between the minimum rate measurement after oligomycin injection and non-mitochondrial respiration). Reported data are the mean values ±SEM of three measurements. ∗ p value <0.05. (E) SK-NBE CTRL and ΔNAGLU clones were grown on coverslips and incubated with MitoTracker. Nuclei (blue) were decorated by DAPI staining. Single focal sections are shown. Images are representative of three independent experiments made in triplicates. Scale bar: 50 μm.

Surprisingly, lactate, succinate, and 3-hydroxyglutarate were the most accumulated metabolites in the MPS IIIB diseased cell system, suggesting an anaerobic glycolytic metabolism and a block of the Krebs cycle in accordance with our previous findings.^10^ Thus, in order to confirm the defect in glycolytic anaerobic metabolism and impairment of mitochondrial activity, we analyzed the metabolic profile of NAGLU silenced SK-NBE, by monitoring the oxygen consumption rate (OCR) profile through the Seahorse system (Seahorse Bioscience, North Billerica, MA, USA) (Figure 6A).

The assay was performed under basal conditions or in the presence of oligomycin (an ATP synthase inhibitor^51^), carbonyl cyanide-4-(trifluoromethoxy) phenylhydrazone (FCCP) (a mitochondrial protonophore uncoupler^52^), as well as rotenone plus antimycin A (two mitochondrial transport chain inhibitors^53^^,^^54^) (Figure 6B). Pharmacological treatment with inhibitors was used to discriminate basal and ATP-linked oxygen consumption rates (OCR). As shown in Figures 6C and 6D, OCR was extremely reduced in the MPS IIIB diseased clone as well as ATP production, clearly indicating and confirming that the MPS IIIB cell model system preferentially uses an anaerobic glycolytic metabolism to survive, as already suggested by the metabolome analyses.

Although the MPS IIIB clone did not show increased proton leakage (Figure 6D), the decreased OCR can indicate an impairment of mitochondrial activity. Thus, to analyze whether the mitochondria of the MPS IIIB clone were morphologically altered, we used the fluorescent dye MitoTracker to stain mitochondria outer membrane. We found out that the mitochondrial outer membranes of the MPS IIIB clone was not damaged; nevertheless, the mitochondria appear to be round-shaped and fragmented compared to the fused and elongated mitochondria of the control clone (CTRL) (Figure 6E). This last experiment further confirmed that the mitochondrial function is severely impaired in the MPS IIIB cell model system.

Overall, these data indicated that the MPS IIIB diseased clones have inactive mitochondrial metabolism with a block of the Krebs cycle, and preferentially survive thanks to anaerobic glycolysis even though this is associated with very low production of ATP.

**Figure 7:**
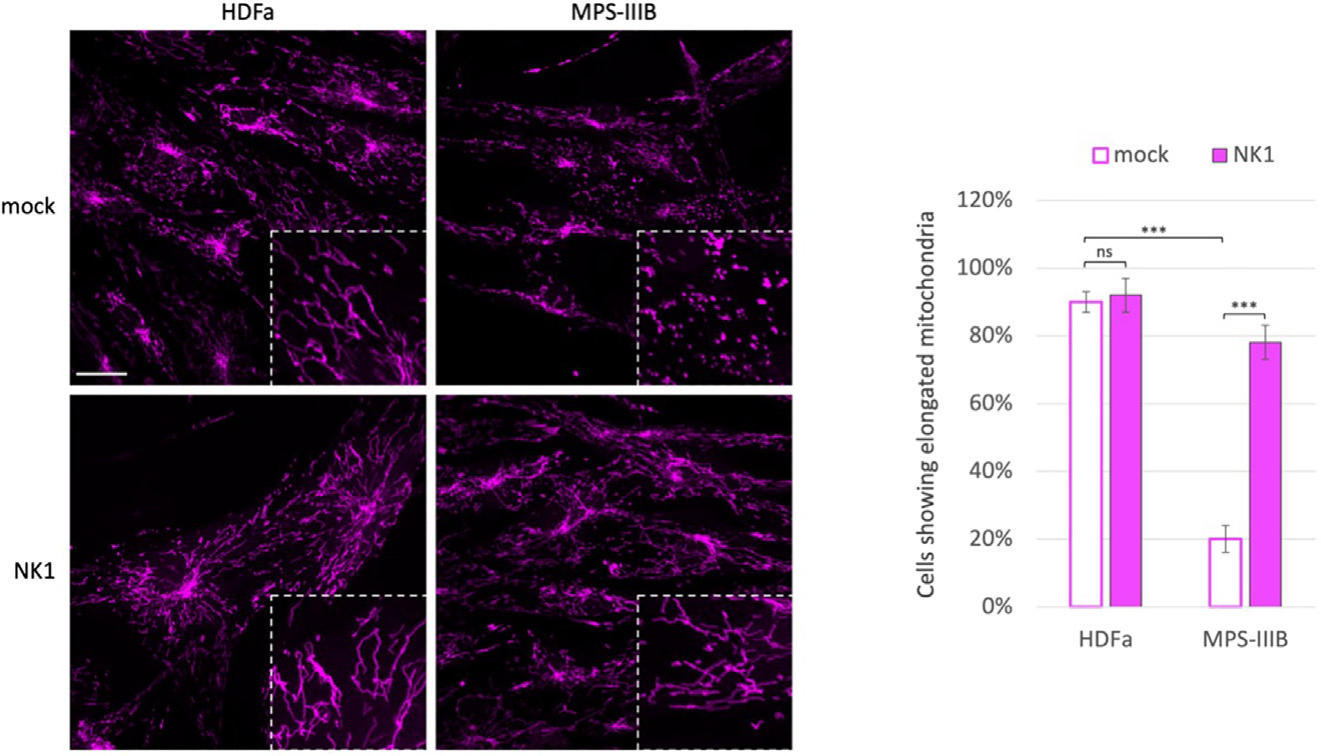
NK1 rescues mitochondrial morphology in MPS IIIB patient fibroblasts Control HDFa and MPS IIIB patient fibroblasts were grown on coverslips and treated or not (mock) with NK1 10^−6^ M for 48 h before being incubated with MitoTracker. Single focal sections are shown. Images are representative of three independent experiments made in triplicates. Scale bar: 50 μm. The histograms on the right show the quantification relative to the percentage of cells showing elongated mitochondria as mean value of fluorescence intensity (pink bars). Means ± SEM were obtained from three independent experiments. ∗∗∗ p value <0.001. ns = not significant.

Since it is common to observe mitophagy processes associated with fragmented mitochondrial morphology in MPS III,^55^^,^^56^^,^^57^ to visualize this correlation also in MPS IIIB fibroblasts we performed MitoTracker on control HDFa and MPS IIIB patient fibroblasts both untreated or treated with NK1 at the concentration of 10^−6^M for 48 h (Figure 7). The results not only confirmed that also in MPS IIIB patient fibroblast the mitochondria are fragmentated, but further confirmed that NK1 was able to restore mitochondrial morphology in MPS IIIB-affected patient fibroblasts.

**Figure 8:**
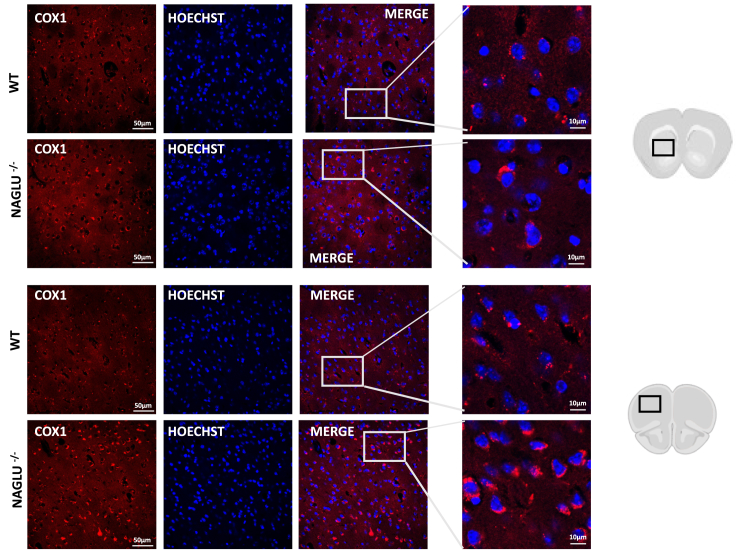
Mitochondrial network show signs of disorganization in striatal (upper panels) and cortical (lower panels) regions of NAGLU^−/−^ mice Forty μm-thick coronal brain sections of 8-month-old wild type (WT) and NAGLU^−/−^ mice were stained with the mitochondrial marker mitochondrially encoded cytochrome *c* oxidase I (MT-CO1, cytochrome *c* oxidase subunit 1 COX1). Nuclei were stained with Hoechst.

Finally, the mitochondrial defects were also analyzed *in vivo* in the brain of MPS IIIB mice by performing an immunofluorescence assay with the mitochondrially encoded cytochrome *c* oxidase I (MT-CO1, cytochrome *c* oxidase subunit 1 COX1). The results are represented in Figure 8 where also MT-CO1 (COX1) staining revealed that the mitochondrial network is deregulated in brain tissues of the MPS IIIB mutant mice suggesting that impaired mitophagy occurs in MPS IIIB mouse brain.

### Inhibition of AMPK rescues HS and lysosome accumulation in MPS IIIB cell model systems

**Figure 9:**
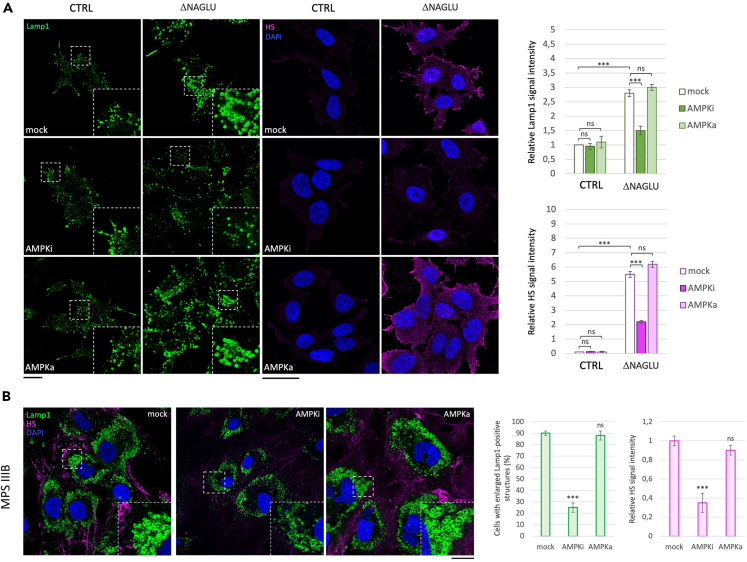
AMPK inhibition rescues lysosomal defects and HS accumulation in MPS IIIB cell model systems (A) SK-NBE (CTRL) and ΔNAGLU clones were grown on coverslips and treated with SBI-0206965, a specific AMPK inhibitor (AMPKi), and 5-aminoimidazole-4-carboxamide ribonucleoside (AICAr), a AMPK allosteric activator (AMPKa), for 24 h before being processed for indirect immunofluorescence. The lysosomal marker LAMP1 (green) and heparan sulfate (pink) proteins were revealed by using specific antibodies. Nuclei (blue) were decorated by DAPI staining. Single focal sections are shown. Images are representative of three independent experiments made in triplicates. Scale bar: 50 μm. (B) MPS IIIB fibroblasts were treated with SBI-0206965 and processed as in A. Single focal sections are shown. Images are representative of three independent experiments made in triplicates. Scale bar: 50 μm. The histograms on the right for (A) and (B) show the quantification relative to the percentage of cells with pathological enlarged lysosomes (green bars) and HS means fluorescence intensity (pink bars). Means ± SEM were obtained from three independent experiments. ∗∗∗ p value <0.001. ns = not significant.

Since the metabolic analyses highlighted low levels of ATP due to hypoxic anaerobic glycolysis, we evaluated whether modulating the AMP-activated protein kinase (AMPK) activity, which impacts both autophagy mechanisms and metabolism,^47^^,^^58^^,^^59^ would have an effect on the MPS phenotype in our cellular model systems. Thus, two specific drugs were tested to assess the involvement of AMPK activity in ΔNAGLU clone. We used SBI-0206965, which occupies a pocket that partially overlaps with ATP-binding site, thus inhibiting the kinase activity of AMPK,^60^ and 5-aminoimidazole-4-carboxamide ribonucleoside (AICAr), a riboside that mimics the effect of AMP on the allosteric activation of AMPK.^61^ LAMP1 and HS immunofluorescence signals were evaluated in ΔNAGLU clone after 24 h of treatment with SBI-0206965. As shown in Figure 9A, AMPK inhibitor (AMPKi) triggered a strong reduction of HS accumulation and lysosomal vacuolization, indicating that the inhibition of autophagy and the metabolic rewiring, through AMPK modulation, could exert a therapeutic action on the MPS IIIB diseased cellular model. By contrast, treatment with the AMPK activator (AMPKa) did not cause any reduction of the lysosomal vacuolization and HS accumulation in the diseased clone (Figure 9A). We also tested the efficacy of the AMPK inhibition on the MPS IIIB patient-derived fibroblasts confirming the same effects of AMPK modulation (Figure 9B).

Finally, in order to dissect the molecular pathways of PIK3/Akt and AMPK we performed western blotting of both Akt and AMPK phosphorylation without and with NK1 treatment in MPS IIIB fibroblasts. According to our previous experiments, Akt resulted phosphorylated (activated) upon NK1 treatment and AMPK dephosphorylated (deactivated) (Figure S7 panel A). These results indicate that, posterior to NK1 binding to the membrane accumulated HS, a signal cascade pathway starts going through the activation of PI3K/Akt pathway and the inhibition of the AMPK signals.

Moreover, we verified whether this mechanism of action would exert an effect on the lysosomal autophagic protein levels. To this aim we performed western blotting analyses to evaluate LAMP1 (lysosomal marker) and BCN1 and LC3 (autophagosome formation and maturation markers) proteins without and with NK1 treatment in MPS IIIB fibroblasts. The western blotting showed that NK1 is able to reduce BCN1 protein levels resulting in both reduction of LC3 and LAMP1 protein levels (Figure S7 panel B) having a curative effect in reducing both lysosomal and autophagy markers.

### Conclusions

Although multiple therapeutic strategies have been explored for the treatment of MPS IIIB, there are still no approved disease modifying treatments and hence significant unmet need to address this devastating progressive neurometabolic disease. Indeed, medical management for individuals with MPS IIIB remains only supportive, serving to alleviate some symptoms, but, do not address the pathogenic mechanisms of the disease.^39^ However, it must also be acknowledged that the molecular mechanisms underlying neurodegeneration in MPS IIIB have not yet been fully elucidated. In this context, we recently demonstrated the ability of NK1, a natural splice variant of HGF, which binds with high affinity the excess of HS accumulated on the cell surface and in the extracellular matrix, to reduce the morphological and functional dysfunctions of lysosomes in primary fibroblasts derived from MPS IIIB affected patients and in a neuronal cellular model of the disease.^4^ Furthermore, we demonstrated that NK1 treatment can restore FGF2-signaling in MPS IIIB patient-derived fibroblasts, thereby reversing deregulated cellular signaling in diseased cells.^7^ We also showed, in our previously published work, that NK1 treatment stimulates the differentiation of NAGLU-silenced SK-NBE into neuron-like cells, thus suggesting that NK1 can reactivate cell signaling involved in the neuronal differentiation process.^4^

In order to develop a new therapeutic strategy for the functional correction of the neurometabolic manifestations of the MPS IIIB disease, based on the use of extracellular HS targeting drugs such as the recombinant NK1 protein, in this study, we explored the ability of NK1 to modulate other fundamental cellular and molecular processes which are commonly associated with dysfunctional lysosomes. Indeed, lysosomes not only are the terminal compartment of both the autophagic and endocytic degradation pathways but also play major roles in instructing cellular homeostasis in response to environmental cues.^12^^,^^33^^,^^34^ For instance, a dysregulation of lysosomal activity may lead to the impairment of various steps of the cellular metabolic machinery, such as the transport of macromolecules (sugars, lipids, proteins, and nucleic acids), and both anabolic and catabolic pathways.^62^

Here, the results of our investigation on the molecular mechanisms underlying the potential therapeutic activity of NK1 in MPS cellular (MPS I, MPS IIIA, MPS IIIB) and animal (MPS IIIB) models for the first time demonstrate that the recombinant NK1 protein: (1) reverts the morphological and positioning alterations of lysosomes in fibroblasts derived from patients affected by MPS I, IIIA, and IIIB by activating PI3K/Akt pathway signaling rather than MEK/ERK pathway; (2) inhibits autophagy through the transcriptional control of the main ALP genes, thus exerting a modulatory activity on autophagosome biogenesis; (3) restores the aerobic glycolytic metabolism and Krebs cycle that are blocked in the diseased cells and tissues. Thus, our results strongly suggest that both the inhibition of autophagy and the metabolic rewiring through AMPK modulation could account for the potential therapeutic activity of NK1 in MPS subtypes where HS accumulates.

In conclusion, our findings provide further insights into mechanisms of MPS IIIB disease and support the beneficial effects of the recombinant protein NK1, thereby opening the way to the development of a novel therapeutic approach for this intractable disease, as well as of other MPS and lysosomal storage diseases.

### Limitations of the study

This study has revealed that upon NK1 treatment occurs the specific reactivation of metabolic pathways that were inactivated in MPS pathological conditions. Moreover, the activation of the PI3K/Akt pathway shed light on its consequential effects on autophagy and metabolic rewiring. While a putative mechanism on the autophagy inhibition and metabolism reactivation through AMPK is discussed in the text, the precise sequences of signaling require further investigations. Moreover, this study has raised new questions about autophagic mechanisms inhibition and metabolism reactivation that need to be elucidated also in *in vivo* models of MPS and other lysosomal storage diseases. In resolving these inquiries, the ongoing research involves the use of the mouse model of the MPS IIIB, followed by a comprehensive study of these mechanisms with a specific focus on the neuropathology of the MPS IIIB disease.

## STAR★Methods

### Key resources table

**Table:** 

REAGENT or RESOURCE	SOURCE	IDENTIFIER
**Antibodies**		

mouse monoclonal anti-CD107a (anti-LAMP1)	Sigma-Aldrich	Cat# SAB4700416; RRID: AB_10932380
mouse monoclonal anti-Heparan Sulfate (10E4)	AMSBIO	Cat# 370255-1 (also 370255-S, NC1183789); RRID: AB_10891554
mouse and rabbit Alexa-Fluor (488 and 546) secondary antibodies	Thermo Fisher Scientific	Cat# A-11029; RRID: AB_2534088, Cat# A-11030; RRID: AB_2737024, Cat# A-11034; RRID: AB_2576217, Cat# A-11035; RRID: AB_2534093
primary mouse anti-activated diphosphorylated ERK1/2 monoclonal antibody	Sigma-Aldrich	Cat# M8159; RRID: AB_477245
rabbit anti-ERK1/2 polyclonal antibody	Promega	Cat# V1141; RRID: AB_430839
mouse anti-phosphorylated Akt monoclonal antibody	Santa Cruz Biotechnology	Cat# sc-514032; RRID: AB_2861344
mouse anti-Akt polyclonal antibody	Santa Cruz Biotechnology	Cat# sc-5298; RRID: AB_626658
rabbit anti-BCN1monoclonal antibody	Santa Cruz Biotechnology	Cat# sc-11427; RRID: AB_2064465
rabbit anti-LC3 polyclonal antibody	Novus Biologicals	Cat# NB100-2331; RRID: AB_10001955
anti-GAPDH monoclonal antibody	Santa Cruz Biotechnology	Cat# sc-32233; RRID: AB_627679
rabbit anti-COX1/MT-CO1 antibody	Cell Signaling Technology	Cat# 62101
goat anti-mouse IgG polyclonal antibody conjugated to horseradish peroxidase (HRP)	Santa Cruz Biotechnology	Cat# sc-2031; RRID: AB_631737
goat anti-rabbit IgG-HRP polyclonal antibody	Santa Cruz Biotechnology	Cat# sc-3837; RRID: AB_650507

**Chemicals, peptides, and recombinant proteins**		

Dulbecco Modified Eagle’s Medium	Thermo Fisher Scientific-Gibco	Cat# 11965092
RPMI-1640 Medium	Thermo Fisher Scientific-Gibco	Cat# 11875093
Fetal bovine serum	Thermo Fisher Scientific-Gibco	Cat# 16000044
Bovine serum albumin	Sigma-Aldrich	Cat# 9048-46-8
PBS, pH7.4	Thermo Fisher Scientific-Gibco	Cat# 10010023
ECL System	Bio-Rad	Cat# 170–5061
Bio-Rad Protein Assay Dye Reagent concentrate	Bio-Rad	Cat# 5000006
Protease inhibitor cocktail tablets	Roche Diagnostics	Cat# 4693116001
Formaldehyde solution 37%	Sigma-Aldrich	Cat# F15587
Methanol	Sigma-Aldrich	Cat# 34860
Trizma base	Sigma-Aldrich	Cat# 1503
Glycine	Sigma-Aldrich	Cat# G8898
Acrylamide/Bis-acrylamide, 30% solution	Sigma-Aldrich	Cat# A3699
ProLongTM Gold Antifade Mountant with DAPI	Thermo Fisher Scientific	Cat# P36935
IBAfect reagent	IBA Lifesciences	Cat# 7-2005-050
MitoTracker™ Red CMXRos	Invitrogen™	Cat# M7512

**Chemicals, peptides, and recombinant proteins**		

Recombinant NK1 fragment of hepatocyte growth factor (HGF)	Pichia pastoris expression system (Pavone LM. et al. Cell Signal. 2011)	N/A
MEK inhibitor PD098059 [2-(2-amino-3-methoxyphenyl)-oxanaphthalen-4-one]	Cayman Chemical	Cat# 10006726
PI3K inhibitor LY294002 [2-(4-morpholinyl)-8-phenyl-4H-1-benzopyran-4-one]	Cayman Chemical	Cat# 70920

**Experimental models: Cell lines**		

Primary Human Dermal Fibroblast from MPSI, MPS IIIA, MPS IIIB	Cell Line and DNA Biobank from Patients Affected by Genetic Diseases (Istituto G.Gaslini, Genova, Italy)	N/A
Primary Human Dermal Fibroblast adult (HDFa)	Thermo Fisher Scientific-Gibco	Cat# C0135C
Human neuroblastoma SK-NBE clones (control clone CTRL and NAGLU silenced clone ΔNAGLU)	Generated in our lab (De Pasquale V. et al. Biochimica et Biophysica Acta Molecular Cell Research 2021)	N/A
HeLa cells mRFP-GFP-LC3B	Kindly provided by prof. David C. Rubinsztein	N/A

**Experimental models: Organisms/strains**		

MPS IIIB (knockout mice, NAGLU^−/−^)	The Jackson Laboratory	Cat# B6.129S6-Naglu^tm1Efn^/J; RRID: IMSR_JAX:003827

**Software and algorithms**		

QuantStudio 6 and 7 Flex	Applied Biosystems	N/A
Fiji ImageJ	National Institutes of Health	N/A
ONE-WAY ANOVA		
MetaboAnalyst 5.0	http://www.metaboanalyst.ca	
MetScape in Cytoscape	http://metscape.ncibi.org	

### Resource availability

#### Lead contact

Further information and requests for resources should be directed to and will be fulfilled by the lead contact, Valeria De Pasquale (valeria.depasquale@unina.it).

#### Materials availability

This study did not generate new unique reagents.

#### Data and code availability

• All data reported in this paper will be shared by the lead contact upon request.
• This paper does not report original code.
• Any additional information required to reanalyze the data reported in this paper is available from the lead contact upon request.

### Experimental model and study participant details

All mouse care and handling procedures were approved by Institutional Animal Care and Use Committee (IACUC) of the Animal Facility of the Department of Molecular Medicine and Medical Biotechnology, University of Naples Federico II (Naples, Italy) (Authorization n° 854/2021-PR from Ministry of Health, Italian Republic). The mouse model of the MPS IIIB (knockout mice, NAGLU^−/−^) were maintained on a 12 h light/dark cycle, identical temperature conditions (21 ± 1°C), humidity (60 ± 5%), and free access to normal mouse chow. The experimental protocols were carried out following ARRIVE guidelines and EU Directive 2010/63/EU for animal experiments.

### Method details

#### Chemicals and reagents

Dulbecco Modified Eagle’s Medium (DMEM), RPMI-1640, fetal bovine serum (FBS), penicillin, streptomycin, and phosphate-buffered saline (PBS) were provided from Gibco, Thermo Fisher Scientific (Carlsbad, CA, USA); ECL System and Bradford assay reagents were from Bio-Rad (München, Germany); protease inhibitor cocktail tablets (4693116001) from Roche Diagnostics (Grenoble, France); bovine serum albumin (BSA) (9048-46-8), formaldehyde solution 37% (F15587), methanol (34860), trizma base (1503), glycine (G8898), acrylamide/bis-acrylamide 30% solution (A3699) from Sigma-Aldrich (St. Louis, MI, USA); ProLongTM Gold Antifade Mountant with DAPI (P36935) from Thermo Fisher Scientific; IBAfect reagent (7-2005-050) from IBA Lifesciences (Goettingen, Germany); MitoTracker Red CMXRos (M7512) from Invitrogen (Carlsbad, CA, USA); MEK inhibitor PD098059 [2-(2′-amino-3′-methoxyphenyl)-oxanaphthalen-4-one] (10006726) and PI3K inhibitor LY294002 [2-(4-morpholinyl)-8-phenyl-4H-1-benzopyran-4- one] (70920) from Cayman Chemical (Ann Arbor, MI, USA).

#### Antibodies

The following antibodies were used for western blotting and immunofluorescence analysis: mouse monoclonal anti-CD107a (anti-LAMP1) (SAB4700416, clone H4A3) from Sigma-Aldrich, mouse monoclonal anti-Heparan Sulfate (10E4) from AMSBIO (370255-1), mouse and rabbit Alexa-Fluor (488 and 546) secondary antibodies (A-11029, A-11030, A-11034, A-11035) from Thermo Fisher Scientific-Invitrogen, (Carlsbad, CA, USA). For Western blot analysis, mouse anti-activated diphosphorylated ERK1/2 monoclonal antibody (M8159) was purchased from Sigma Aldrich Chemical Co., rabbit anti-ERK1/2 polyclonal antibody (V1141) from Promega (USA), mouse anti-phosphorylated Akt monoclonal antibody (sc-514032) was purchased from Santa Cruz Biotechnology (Heidelberg, Germany), mouse anti-Akt polyclonal antibody (sc-5298) Santa Cruz Biotechnology (Heidelberg, Germany), rabbit anti-BCN1 monoclonal antibody (H-300 sc-11427) was purchased from Santa Cruz Biotechnology (Heidelberg, Germany), rabbit anti-LC3 polyclonal antibody (NB100-2331) from Novus Biologicals (USA), mouse anti-GAPDH monoclonal antibody (6C5 sc-32233) from Santa Cruz Biotechnology (Heidelberg, Germany), rabbit anti-COX1/MT-CO1 antibody (62101) from Cell Signaling Technology (Danvers, Massachusetts, USA), whereas the secondary antibodies goat anti-mouse IgG polyclonal antibody conjugated to horseradish peroxidase (HRP) (sc-2031) and goat anti-rabbit IgG-HRP polyclonal antibody (sc-3837) were from Santa Cruz Biotechnology (Heidelberg, Germany).

#### Cell culture and treatments

The fibroblasts from MPS-affected patients (MPS I, MPS IIIA, MPS IIIB) used in this study, their genotypes and phenotypes of the original patients, were kindly provided by the Cell Line and DNA Biobank from Patients Affected by Genetic Diseases (Istituto G. Gaslini, Genoa, Italy).^63^ MPS fibroblast characteristics: MPS I genotype p.W402X/p.W402X with severe phenotype; MPS IIIA genotype c.1079delC/c.1079delC with intermediate phenotype; MPS IIIB genotype p.V501G/p.V501G with severe phenotype.

Primary Human Dermal Fibroblasts adult (HDFa) from Gibco, and MPS fibroblasts were cultured in DMEM, supplemented with 10% FBS, 2 mM L-glutamine, 100 U/ml penicillin, and 100 mg/mL streptomycin, at 37°C in a humidified 5% CO_2_ atmosphere.

SK-NBE human neuroblastoma clones (control clone CTRL, and NAGLU silenced clone ΔNAGLU) were cultured in RPMI-1640, 2 mM L-glutamine, 1 mM sodium pyruvate, supplemented with 10% FBS, 100 U/mL penicillin, and 100 μM/mL streptomycin, supplemented with 0.7 μM/mL of puromycin at 37°C in a humidified 5% CO_2_ atmosphere.

SK-NBE clones and MPS IIIB fibroblasts were first grown under normal conditions at 80% confluence, kept in serum deprivation for 6 h, and then treated with Beclin1 siRNA (siRNA BCN1, Invitrogen) by cell transfection system IBAfect (IBIDI) according to manufacturer’s instructions.

HeLa cells mRFP-GFP-LC3B were cultured in DMEM, supplemented with 10% FBS, 2 mM L-glutamine, 100 U/mL penicillin, and 100 μg/mL streptomycin, at 37°C in a humidified 5% CO_2_ atmosphere.

#### Fluorescence microscopy

Immunofluorescence staining was performed as previously reported.^64^^,^^65^^,^^66^ Briefly, cells (SK-NBE clones, HDFa and MPS-derived patient fibroblasts) grown on glass coverslips were washed with PBS and fixed in 3.7% formaldehyde at room temperature for 30 min. After fixation, cells were washed with PBS and permeabilized by incubation in blocking buffer (PBS containing 1% BSA, 0.01% sodium azide, and 0.02% Saponin) for 10 min at room temperature. Cells were then incubated with the indicated primary antibody diluted in the same blocking buffer for 1 h at room temperature. Cells were washed three times with PBS and incubated with the corresponding secondary antibody for 30 min at room temperature. Finally, coverslips were washed in distilled water and mounted onto glass slides with the Prolong Gold anti-fade reagent with DAPI. SK-NBE clones were treated with medium containing 50 nM MitoTracker Red CMXRos (M7512, Invitrogen) to visualize mitochondria morphology and distribution. Images were collected using a laser-scanning microscope (LSM 700, Carl Zeiss Microimaging, Inc., Jena, Germany) equipped with a planapo 63× oil immersion (NA 1.4) objective lens.

#### Western blotting

Cells, grown to sub-confluence in standard medium, were harvested in lysis buffer (50 mM Tris pH 7.5, 150 mM NaCl, 1 mM EDTA, 1 mM EGTA, 10% glycerol, 1% Triton X-100, 1 mM β-glycerophosphate, 1 mM phenylmethylsulfonyl fluoride, protease inhibitor cocktail tablet, 1 mM sodium orthovanadate, and 2.5 mM sodium pyrophosphate).^67^ The lysates were incubated for 30 min on ice, and supernatants were collected, and centrifuged for 30 min at 13000 rpm. Protein concentration was estimated by Bradford assay, and 25 or 50 μM/lane of total proteins were separated on SDS gel and transferred to nitrocellulose membrane. Membranes were treated with a blocking buffer (25 mM Tris, pH 7.4, 200 mM NaCl, 0.5% Triton X-100) containing 5% nonfat powdered milk for 1 h at room temperature. Incubation with the primary antibody was carried out overnight at 4°C. After washings, membranes were incubated with the HRP-conjugated secondary antibody for 1 h at room temperature. Following further washings of the membranes, chemiluminescence was generated by enhanced chemiluminescence (ECL) kit.

#### Seahorse analysis of oxygen consumption rate (OCR)

The real-time oxygen consumption rate (OCR) of SK-NBE clones (CTRL and ΔNAGLU) was measured at 37°C using a Seahorse XF Analyzer (Seahorse Bioscience, North Billerica, MA, USA). SK-NBE clones were plated into specific cell culture microplates (Agilent, Santa Clara, CA, USA) at the concentration of 3x10^4^ cells/well, and cultured for 12 h in DMEM, 10% FBS. OCR was measured in XF media (non-buffered DMEM medium, containing 10 mM glucose, 2 mM L-glutamine, and 1 mM sodium pyruvate) under basal conditions and after sequential addition of 1.5 μM oligomycin, 2 μM FCCP, and rotenone + antimycin (0.5 μM all) (all from Agilent). Indices of mitochondrial respiratory function were calculated from the OCR profile: basal OCR (before addition of oligomycin), maximal respiration (calculated as the difference between FCCP rate and antimycin + rotenone rate), spare respiratory capacity (calculated as the difference of FCCP-induced OCR and basal OCR), ATP production (calculated as difference between basal OCR and oligomycin-induced OCR) and proton leak (calculated as the difference between the minimum rate measurement after oligomycin injection and non-mitochondrial respiration). Reported data were the mean values ± SEM of three measurements deriving from two independent experiments.

#### Mouse model

The animal model of MPS IIIB (knockout mice, NAGLU^−/−^) were generated by Prof. Elizabeth Neufeld, UCLA, by insertion of neomycin resistance gene into exon 6 of NALGU gene on the C57/BL6 background.^29^ NAGLU knockout mice (NAGLU^−/−^) available to us were genotyped as previously described.^5^ Mice (4 per cage) were maintained on a 12 h light/dark cycle, identical temperature conditions (21 ± 1°C), humidity (60 ± 5%), and free access to normal mouse chow. The experimental protocols were carried out following ARRIVE guidelines and EU Directive 2010/63/EU for animal experiments. All mouse care and handling procedures were approved by Institutional Animal Care and Use Committee (IACUC) of the Animal Facility of the Department of Molecular Medicine and Medical Biotechnology, University of Naples Federico II (Naples, Italy) (Authorization n° 854/2021-PR from Ministry of Health, Italian Republic). The sacrifice of the NAGLU^+/+^ and NAGLU^−/−^ mice was performed in the morning to avoid sample collection variation due to time. The whole brain was rapidly removed, washed with ice-cold PBS, and stored at −80°C for the metabolomic studies.

#### Metabolite extraction and derivatization

Metabolites were identified and quantified from collected cells and whole brain tissues using a targeted mass spectrometry (MS)-based platform.^68^ The frozen tissues and cellular pellets were homogenized in 85:15 cold ethanol/0.1M phosphate buffer (ratio 1:6 w/v and 1:2 v/v for tissues and cellular pellets, respectively), using a TissueLyser LT homogenizer, (Qiagen, Duesseldorf, Germany). The mixtures were centrifuged at 13000 rpm, 30 min, 4°C, to collect the supernatant for metabolome analysis. The protein pellets were solubilized in lysis buffer (7 M urea, 2 M thiourea, 30 mM Tris-HCl, and 4% CHAPS) to estimate the protein content.

Aliquots of samples, corresponding to 50 μM of proteins, were transferred onto a 96-well plate containing the positions for blanks, PBS, calibrants, and quality controls (QC) according to the protocols of MxP Quant 500 kit (Biocrates Life Sciences AG, Innsbruck, Austria).^69^ The mixtures were dried under nitrogen stream, and then incubated in 50 μL of 5% phenyl isothiocyanate (PITC) for 1 h for further metabolite extraction in 5 mM ammonium acetate in methanol. The extracted mixtures were analyzed by LC-MS/MS in multiple reaction monitoring (MRM) mode using an SCIEX Triple Quad System 5500+ QTRAP Ready (AB Sciex, Framingham, MA, USA) coupled to a 1260 Infinity II HPLC (Agilent). MS data were processed using the Analyst software v.1.7.1 (AB Sciex, Framingham, MA, USA) and the MetIDQ Oxygen software (Biocrates Life Sciences AG, Innsbruck, Austria) to integrate targeted metabolite peaks for accurate quantification. The metabolomic analysis allowed to target 106 metabolites, including amino acids (AA) (20 molecules), AA related (30 molecules), bile acids (14 molecules), fatty acids (12 molecules), biogenic amines (9 molecules), carboxylic acids (7 molecules), hormones (4 molecules), indoles derivatives (4 molecules), and alkaloids, amine oxides, cresols, vitamins and cofactors (6 molecules).^70^

#### Metabolite feature selection

The metabolome datasets were processed by chemometrics and cluster methods using MetaboAnalyst 5.0 (http://www.metaboanalyst.ca).^71^ The features with more than 50% of null values were removed, whereas missing values were replaced by 1/5 of the minimum positive value of each hit in the dataset. Abundance values were then log10-transformed and Pareto-scaled. The Principal Component Analysis (PCA) and Partial Least Squares Discriminant Analysis (PLS-DA) were performed to find the variance in the datasets and predict the class of relevant features by multivariate regression techniques, respectively. To this aim, VIP (Variable Importance in Projection) metabolites were retrieved to show the importance of each analyte to predict diverse conditions (VIP score >1). Hierarchical cluster analysis was performed to rank by t-test (p < 0.05) in a heatmap the compared groups according to their relative metabolite abundances. Univariate statistical analysis was carried out by GraphPad Prism 9.0.^72^^,^^73^ Volcano plots were built to select significantly varying abundances (–log10(q-value) >2), calculated as ratio of metabolite levels with respect to control levels (fold change). The significant differences for multiple comparisons of single molecules were evaluated by ordinary one-way ANOVA for normally distributed data or non-parametric Kruskal-Wallis test, coupled with multiple comparison corrections. The significant differences for binary comparisons of single molecules were evaluated by parametric Welch’s t-test or non-parametric Mann-Whitney t-test. The normal distribution was verified according to D'Agostino and Pearson tests.

#### Metabolome functional enrichment analysis

The enrichment analysis of representative metabolic pathways in ΔNAGLU and NAGLU^−/−^ mice was carried out using the MetScape app in Cytoscape software v3.9.1.^74^ A compound-compound network type was built, selecting human and mouse as organisms and important metabolites as input. Compounds names or their KEGG identification codes were used to map the metabolic pathways.

#### Tissue processing, immunostaining, and confocal immunofluorescence

NAGLU +/+and NAGLU^−/−^ mice were anesthetized and transcardially perfused with saline solution containing 0.01 mL heparin, followed by 4% paraformaldehyde in 0.1 mol/L PBS saline solution. Brains were processed as previously described.^75^ Briefly, brains were rapidly removed on ice and postfixed overnight at + 4°C and cryoprotected in 30% sucrose in 0.1 M phosphate buffer (PB) with sodium azide 0.02% for 24 h at 4°C. Brains were sectioned frozen on a sliding cryostat at 40 μm thickness, in rostrum-caudal direction. Afterward, free floating serial sections were incubated with PBS Triton X-0.3% and blocking solution (0.5% milk, 10% FBS, 1% BSA) for 1 h and 30 min. The sections were incubated overnight at + 4°C with the primary antibody anti-COX1. The sections were then incubated with the corresponding florescent-labeled secondary antibodies, Alexa 488/Alexa 594 conjugated antimouse/antirabbit IgG. Nuclei were counterstained with Hoechst. Images were observed using a Zeiss LSM700 META/laser scanning confocal microscope (Zeiss, Oberkochen, Germany). Single images were taken with a resolution of 1024 × 1024.^76^

### Quantification and statistical analysis

Data reported are expressed as the mean ± SD of at least three separate experiments. Statistical significance was determined by Student’s *t*-test and ONE-WAY ANOVA test. A value of p < 0.05 was considered statistically significant.

## Supplemental information

Document S1. Figures S1–S7

Table S1. Metabolite abundance (µM) in MPS IIIB cellular model

Table S2. Metabolite abundance (µM) in MPS IIIB mouse model brains
