# Supplementary material for: Metabolic rewiring and autophagy inhibition correct lysosomal storage disease in mucopolysaccharidosis IIIB
Source: iScience. 2024 Jan 29;27(3):108959. doi: 10.1016/j.isci.2024.108959 (PMC10864807; doi:10.1016/j.isci.2024.108959)
Supplement: Document S1. Figures S1–S7 [file mmc1.pdf]

iScience, Volume *27*

Supplemental information

Metabolic rewiring and autophagy inhibition correct lysosomal storage disease in mucopolysaccharidosis IIIB

Melania Scarcella, Gianluca Scerra, Mariangela Ciampa, Marianna Caterino, Michele Costanzo, Laura Rinaldi, Antonio Feliciello, Serenella Anzilotti, Chiara Fiorentino, Maurizio Renna, Margherita Ruoppolo, Luigi Michele Pavone, Massimo D'Agostino, and Valeria De Pasquale

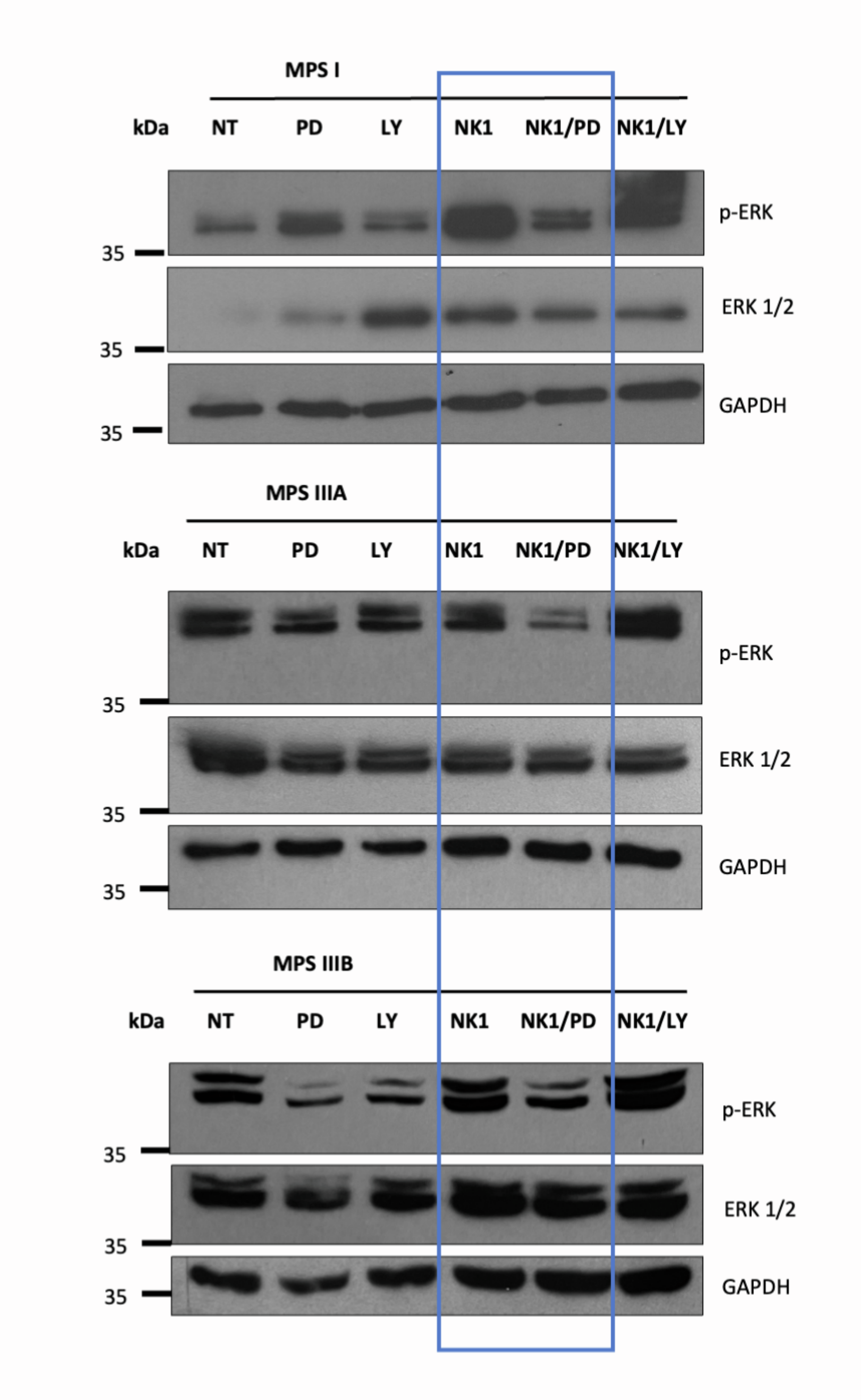

Figure S1. p-ERK protein levels in MPS fibroblasts as detected by Western blotting analysis, related to Figure 2

Patient fibroblasts untreated and treated with PD98059, LY294002, NK1, NK1/PD98059 and NK1/LY294002 were analyzed for ERK1/2 phosphorilation with specific anti-p-ERK, anti-ERK antibodies. Anti-GAPDH antibody was used to ensure equal protein loading in all gel lanes. The blots reported are representative of three independent experiments of equal design.

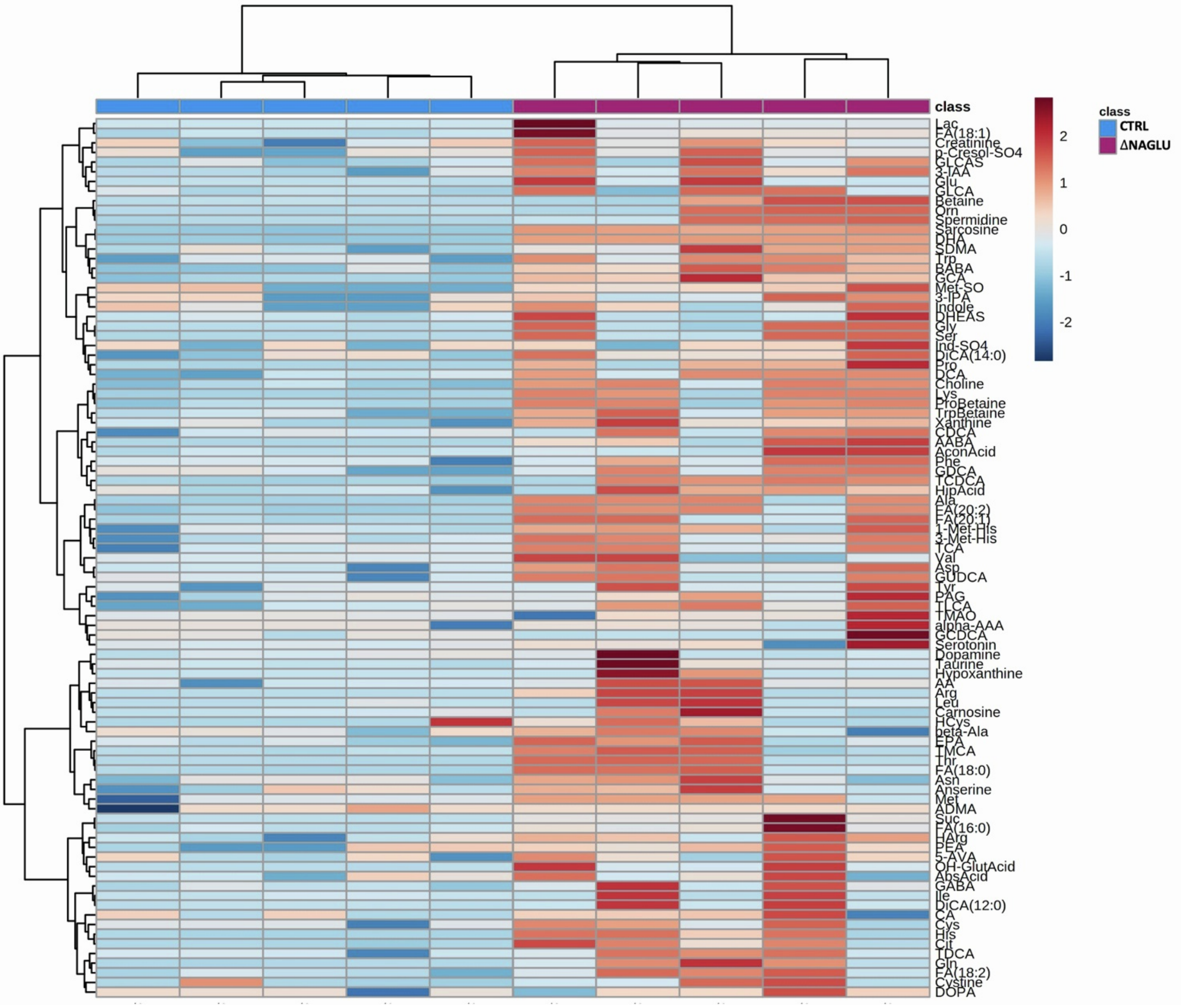

Figure S2. Heatmap of cellular metabolites concentrations in each group (ΔNAGLU vs CTRL), ranked by t-test ($p<0.05$), related to Figure 5

The intensity of the colored boxes represents the relative abundance in each group. The concentrations (μM) of the identified metabolites were normalized by total protein content (50 μg), log10-transformed, and Pareto-scaled.

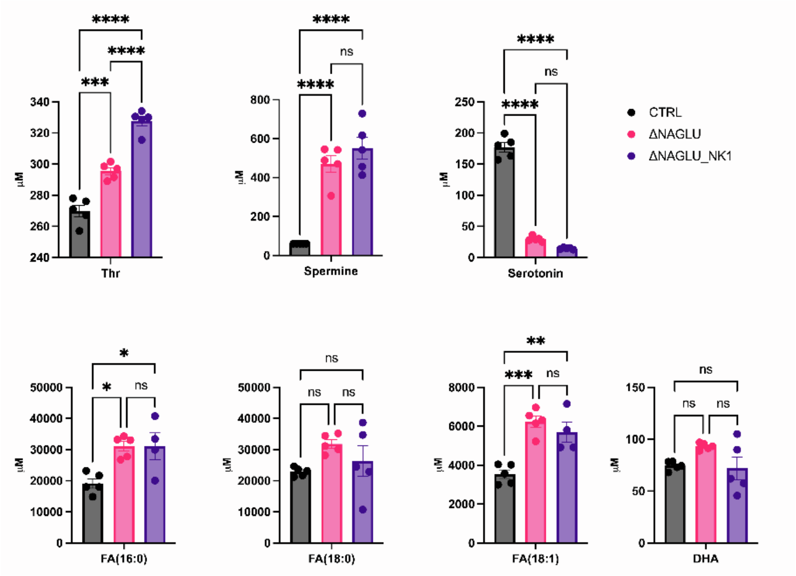

Figure S3. Abundance of discriminant metabolites in ΔNAGLU were evaluated in comparison with CTRL and ΔNAGLU treatment with NK1 (ΔNAGLU_NK1), related to Table 1

Plots represents the analytes concentrations (means ± SEM). The significant differences between groups were evaluated performing ordinary one-way ANOVA test and Hold-Sidak's multiple comparison test in normally distributed datasets or Kruskal-Wallis test and Dunn's multiple comparison test in not-normally distributed datasets. The normal distribution was verified according to D'Agostino and Pearson test. (* $p<0.05$, ** $p<0.01$, *** $p<0.001$ **** $p<0.0001$, ns = not significant).

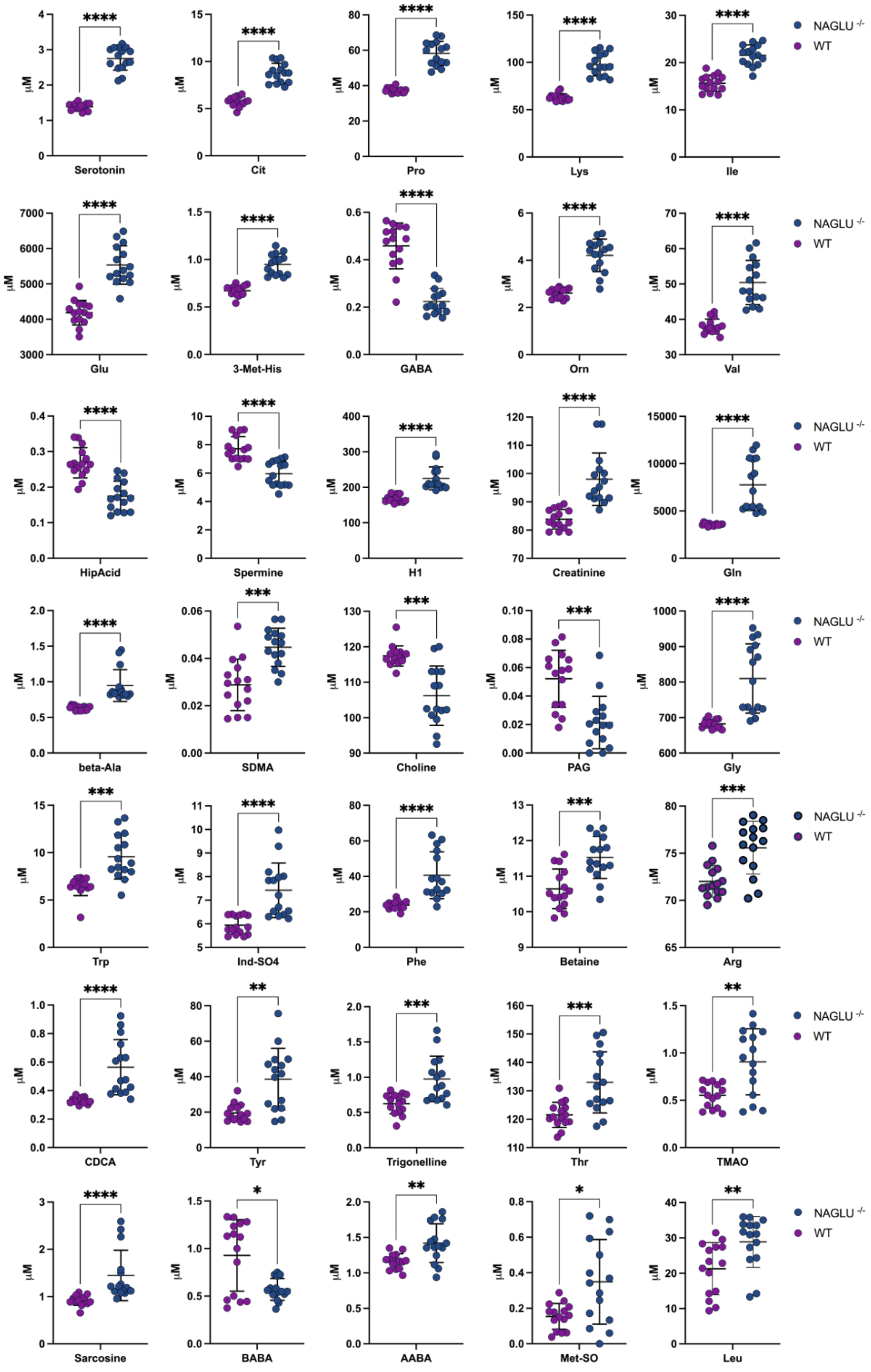

Figure S4. Abundances of discriminant metabolites in brain tissue from NAGLU$^{-/-}$ mice were evaluated as compared to WT, related to Table 2

Plots represents the analytes concentrations (means ± SEM). The significant differences between groups were evaluated performing parametric t test with Welch correction in normally distributed datasets or Mann-Whitney test in not-normally distributed datasets (* p<0.05, ** p<0.01, *** p<0.001 **** p<0.0001, ns = not significant).

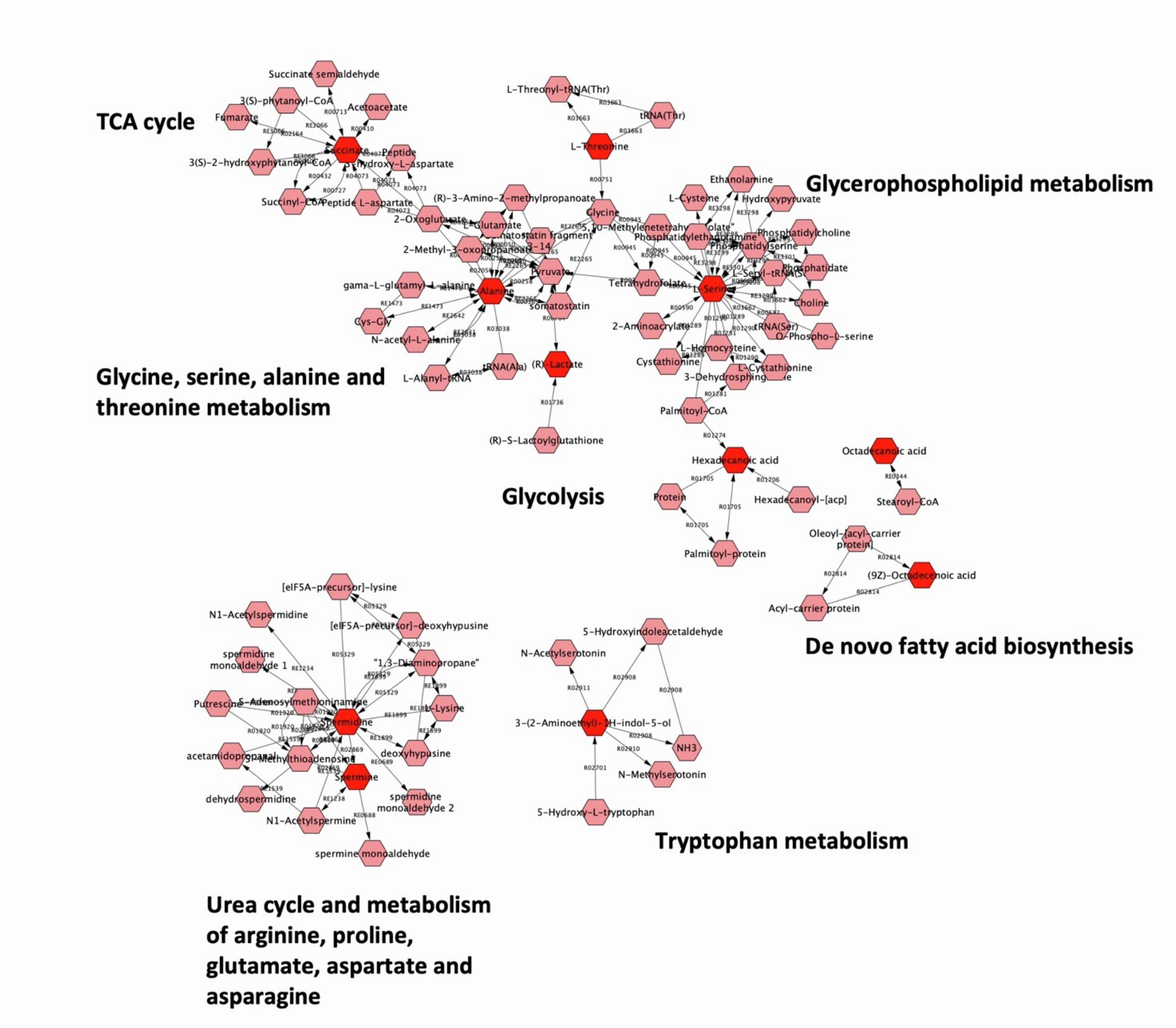

Figure S5. Relevant metabolic networks and enriched pathways obtained from the differential metabolome of ΔNAGLU cells, related to Figure 5

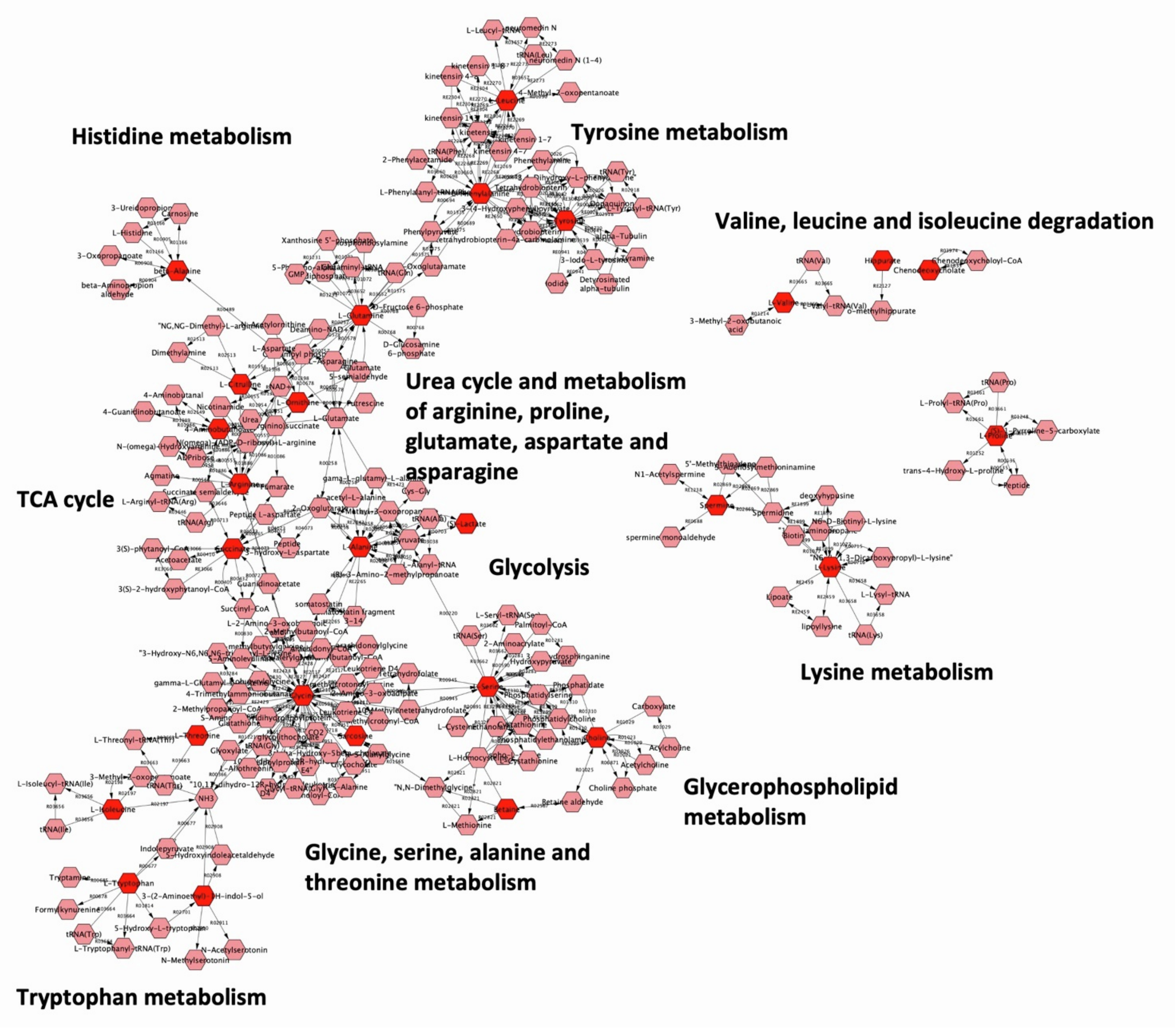

Figure S6. Relevant metabolic networks and enriched pathways obtained from the differential metabolome of NAGLU$^{-/-}$ mice brains, related to Figure 5

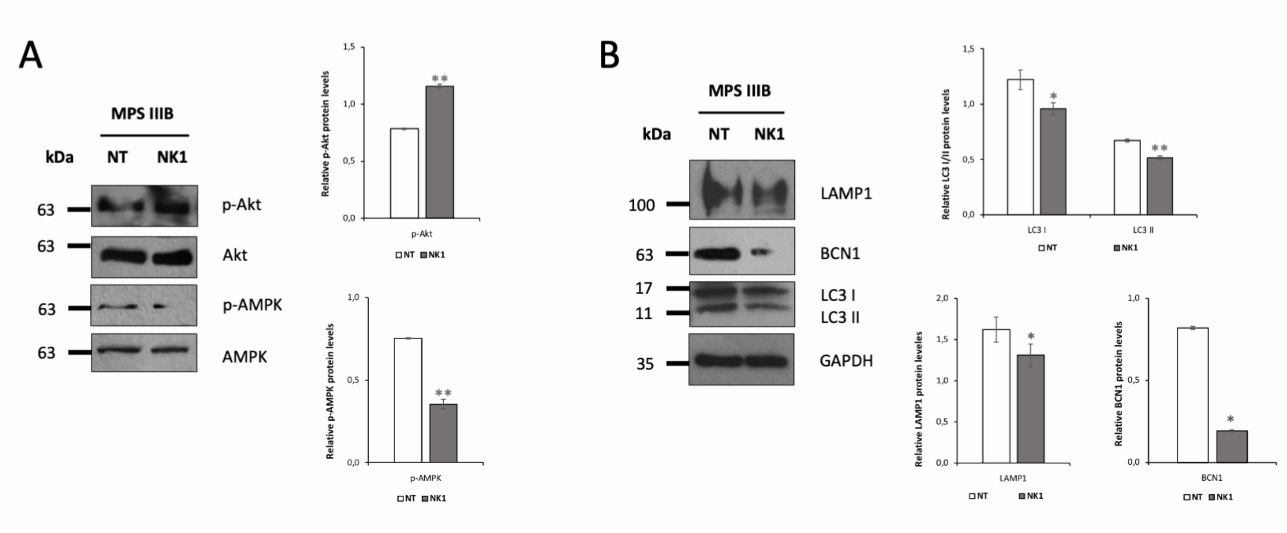

Figure S7. Molecular analysis of the signaling activated by NK1 in MPS IIIB fibroblasts and the impact on the autophagic proteins, related to Figure 2, 3, 4 and 9

(A) p-Akt, p-AMPK protein levels in MPS fibroblasts untreated and treated with NK1 10^{-6}M as detected by Western blotting analysis. Membranes were incubated with anti-p-Akt, anti-Akt, anti-p-AMPK, anti-AMPK and anti-GAPDH antibodies was used to ensure equal protein loading in all gel lanes. The blots reported are representative of three independent experiments of equal design. Densitometric analysis of the bands was performed, and the data obtained are reported on histograms. **P < 0.01.

(B) LAMP1, BCN1, LC3 I, II protein levels in MPS fibroblasts untreated and treated with NK1 10^{-6}M as detected by Western blotting analysis. Membranes were incubated with anti-LAMP1, anti-BCN1, anti-LC3 and anti-GAPDH antibodies was used to ensure equal protein loading in all gel lanes. The blots reported are representative of three independent experiments of equal design. Densitometric analysis of the bands was performed, and the data obtained are reported on histograms. *P < 0.05, **P < 0.01.
